# Mapping in silico genetic networks of the *KMT2D* tumour suppressor gene to uncover novel functional associations and cancer cell vulnerabilities

**DOI:** 10.1186/s13073-024-01401-9

**Published:** 2024-11-22

**Authors:** Yuka Takemon, Erin D. Pleasance, Alessia Gagliardi, Christopher S. Hughes, Veronika Csizmok, Kathleen Wee, Diane L. Trinh, Ryan D. Huff, Andrew J. Mungall, Richard A. Moore, Eric Chuah, Karen L. Mungall, Eleanor Lewis, Jessica Nelson, Howard J. Lim, Daniel J. Renouf, Steven JM. Jones, Janessa Laskin, Marco A. Marra

**Affiliations:** 1 Genome Science and Technology Graduate Program, University of British Columbia, Vancouver, Canada; 2 Michael Smith Laboratories, University of British Columbia, Vancouver, Canada; 3 Canada’s Michael Smith Genome Sciences Centre, BC Cancer Research Institute, Vancouver, Canada; 4 Department of Medicine, Dalhousie University, Halifax, Canada; 5 Division of Respiratory Medicine, Department of Medicine, Air Pollution Exposure Laboratory, Vancouver Coastal Health Research Institute, University of British Columbia, Vancouver, BC Canada; 6 Department of Medical Oncology, BC Cancer, Vancouver, BC Canada; 7 Pancreas Centre BC, Vancouver, BC Canada; 8 Department of Medical Genetics, University of British Columbia, Vancouver, Canada

**Keywords:** Tumour suppressor genes, *KMT2D*, Genetic networks, Synthetic lethality, WRN inhibitors

## Abstract

**Background:**

Loss-of-function (LOF) alterations in tumour suppressor genes cannot be directly targeted. Approaches characterising gene function and vulnerabilities conferred by such mutations are required.

**Methods:**

Here, we computationally map genetic networks of *KMT2D*, a tumour suppressor gene frequently mutated in several cancer types. Using *KMT2D* loss-of-function (*KMT2D*^LOF^) mutations as a model, we illustrate the utility of in silico genetic networks in uncovering novel functional associations and vulnerabilities in cancer cells with LOF alterations affecting tumour suppressor genes.

**Results:**

We revealed genetic interactors with functions in histone modification, metabolism, and immune response and synthetic lethal (SL) candidates, including some encoding existing therapeutic targets. Notably, we predicted *WRN* as a novel SL interactor and, using recently available WRN inhibitor (HRO761 and VVD-133214) treatment response data, we observed that *KMT2D* mutational status significantly distinguishes treatment-sensitive MSI cell lines from treatment-insensitive MSI cell lines.

**Conclusions:**

Our study thus illustrates how tumour suppressor gene LOF alterations can be exploited to reveal potentially targetable cancer cell vulnerabilities.

**Supplementary Information:**

The online version contains supplementary material available at 10.1186/s13073-024-01401-9.

## Background

Loss-of-function (LOF) alterations in tumour suppressor genes are conceptually challenging to target therapeutically, due to the reduction or complete loss of the encoded protein that, under normal conditions, provides the cellular restraints necessary to prevent, e.g. aberrant proliferation or genomic instability [1]. The functional inadequacy or complete lack of mutated tumour suppressor gene protein renders most inhibitory drugs illogical in contrast to therapies targeting oncogenic proteins [1]. Genetic networks, namely essentiality networks [2–6] and genetic interaction (GI) networks [7–9], provide a powerful means to map and interrogate functional interaction landscapes and to identify vulnerabilities of cells harbouring LOF alterations in tumour suppressor genes. Briefly, essentiality networks include co-essential and anti-essential genes, where the perturbation of two genes has similar (synergistic) or opposing (antagonistic) effects on cell survival across various cell lines, respectively [2, 4, 5]. GI networks include synthetic lethal (SL) and alleviating (AL) interactors, where the perturbation of two genes together in the same cell confers reduced or advantageous fitness effects, respectively, but the perturbation of either gene alone does not affect cell fitness [7]. Genetic network analyses have revealed novel functional relationships between genes, pathways, and cancer cell vulnerabilities [2–6, 8, 9]. Even so, mapping genetic networks at scale using in vitro screening techniques remains a challenge [10, 11].

Here, we report the use of an in silico genetic screening approach to systematically characterise tumour suppressor gene function and vulnerabilities of cancer cells harbouring LOF alterations in a tumour suppressor gene. We applied our method to map the genetic networks of *KMT2D*, a frequently mutated tumour suppressor gene across cancer types [12]. Examination of *KMT2D*’s essentiality network revealed novel associations with genes that play roles in histone modification, transcription, mitotic cell cycle regulation, glycolysis, and DNA replication, and those encoding proteins that interact with KMT2D on the chromatin. Through mapping cancer type-specific in silico* KMT2D* GI networks, we revealed several SL candidates, namely *NDUFB4*, *MDM2*, *TUBA1B*, and *WRN*, that encode targets of existing and in-development therapeutics, making them potentially viable drug targets in cancers harbouring *KMT2D*^LOF^ alterations. Using The Cancer Genome Atlas (TCGA) data, we showed dysregulated SL candidate-associated functions, such as in p53 regulation and metabolism. Using data from TCGA, Memorial Sloan Kettering Cancer Center Integrated Mutation Profiling of Actionable Cancer Targets (MSK-IMPACT; NCT01775072 [13]), and the Personalized OncoGenomics (POG) Program at BC Cancer (NCT021556210 [14, 15]), a cohort of advanced and metastatic cancer patients that have been heavily treated; we also showed that MSI cases with *KMT2D*^LOF^ alterations show significantly elevated immune checkpoint response markers compared to *KMT2D*^WT^ MSI cases, indicating *KMT2D*^LOF^ mutations may be a biomarker for immune checkpoint inhibitor (ICI) treatment stratification. Strikingly, compared to *KMT2D*^WT^ cancer cell lines with microsatellite instability (MSI), we show that *KMT2D*^LOF^ MSI cell lines are more sensitive to *WRN* KO and thus, as predicted, to treatment with two recently published WRN inhibitors, which are in phase I clinical trials (NCT05838768 [16] and NCT06004245 [17]). Our work thus models a more general approach, in which in silico genetic network maps are used to identify novel functional associations, cancer cell vulnerabilities, and novel treatment opportunities associated with tumour suppressor gene alterations.

## Methods

### Generation of HEK *KMT2D*^KO^ lines

The human embryonic kidney cell line HEK293A (provided by Dr. Gregg Morin, Genome Sciences Centre, BC Cancer and authenticated by Genetica DNA Laboratories, Cincinnati, OH) was cultured in Dulbecco’s modified Eagle’s medium (DMEM; Life Technologies) supplemented with 10% (v/v) heat-inactivated foetal bovine serum (FBS; Life Technologies) in a 37 °C incubator with 5% CO_2_ humidified atmosphere. Generation of HEK293A *KMT2D* knockout cells (herein referred to as *KMT2D*^KO^; *KMT2D*^KO1^ [D320] and *KMT2D*^KO3^ [D372]) were performed using CompoZr Knockout Zinc Finger Nucleases (CKOZFND14397, Sigma) targeting *KMT2D* exon 39 (Chr12: 49,427,414–49,427,455, NCBI build hg19). Each individual ZFN recognises an 18-bp DNA sequence on either side of a 6-bp spacer sequence where the heterodimerised FokI domains cleave the DNA. In total, the ZFN recognition and cleavage site (underlined) consists of 42 bases:

GGGATCCAGCCCCACCAGAATGTTGCTGTTGCTGCTGTTGGG

These *KMT2D*-specific ZNF plasmids were co-transfected with a Hygro-RS-MLL2 ZNF reporter plasmid (ToolGen) into HEK293A. This reporter plasmid was used to assess ZFN efficiency [18] through the co-expression of the mRFP gene, the ZNF recognition/cleavage sites and an out-of-frame Hygro-eGFP gene, which has a one in three chance of an in-frame repair following ZNF nuclease activity. Forty-eight hours post-transfection, the cells were single-cell sorted based on double RFP + /GFP + expression using the FACSAriaII (BD Biosciences). Single cells were expanded and screened for clones bearing mutation of *KMT2D* in all alleles through immunoblotting, and frameshift mutations were confirmed through sequencing.

### ChIP-MS analysis

#### SILAC and ChIP

For heavy labelling, HEK-*KMT2D*^WT^ cells were grown in Dulbecco’s modified Eagle’s medium (DMEM) deficient in lysine and arginine (Thermo Fisher Scientific) supplemented with dialysed FBS (Thermo Fisher Scientific), MEM Non-Essential Amino Acids (Thermo Fisher Scientific), Lys-8 (CNLM-291-H-0.25, Cambridge Isotope Laboratories), and Arg-10 (CNLM-539-H-0.25, Cambridge Isotope Laboratories) for 6 days before using the cells for ChIP. ChIP-MS was performed as described by Engelen et al. [19] with substitution of physical chromatin fragmentation (by sonication) with enzymatic treatment to preserve the integrity of high molecular weight proteins. Briefly, isolated nuclei were washed twice with 1X MNase Buffer (0.1% Na-Deoxycholate, 0.1%Triton X-100, CEF + NaButyrate) followed by centrifugation at 4 °C for 5 min at 2800 rpm and supernatant discarded. Each pellet was resuspended in 3 pellet volumes of 1X MNase Buffer. Four pellet volumes of 2X MNase digestion buffer containing 1 mM DTT and the MNase enzyme (diluted 1:100 in the total volume) were added. MNase digestion was performed at room temperature for 7 min and then stopped with EGTA to a final concentration of 20 mM. MNase Buffer was added to a final concentration of 1X to help chromatin solubilisation on ice for 15 min. Digested chromatin was resuspended in IP buffer (200 mM NaCl, 1 mM EDTA, 0.15% NaDOC, 1.5% Triton, 0.1% SDS), and samples were sonicated for 15 s (Covaris, Duty 10%—Intensity 4—Burst 200 in 1 ml tubes) to break the nuclei. DNA fragment size was confirmed to be between 150 and 400 bp. Approximately 120 mg chromatin (as measured by Nanodrop at UV absorbance at 280 nm) were used for each sample and extracts from HEK-*KMT2D*^WT^ and HEK-*KMT2D*^KO1^ or HEK-*KMT2D*^KO3^ were mixed at one-to-one ratio. Two independent replicates for each *KMT2D* WT:KO mix underwent chromatin immunoprecipitation. Relative amounts of protein present in each sample were visualised on 10% precast SDS–PAGE gels (NuPage Invitrogen) and stained with colloidal Coomassie stain (Invitrogen) after cross linking reversal in the buffer containing 100 mM NaHCO_2_, 2% SDS and followed by overnight incubation at 68 ℃. The following day the reaction was complemented with 20 mM MgCl_2_ and 10 µl of Benzonase nuclease (Millipore) to remove the DNA. Antibody used in the ChIP was against KMT2D (Sigma Prestige). For each ChIP, 50 μg antibody was crosslinked to 500 μl Protein A magnetic bead solution (15-mg beads, Life Technologies) with Dimethyl Pimelimidate (Sigma) to prevent the interference of immunoglobulin elution with the MS analysis. Crosslinked antibody–bead complexes were equilibrated in the buffer containing 20 mM TrisHCl pH 8, 300 mM NaCl, 2 mM EDTA, 1 mM EGTA, 0.2% NaDOC, 2% Triton X-100, and pre-cleared with 0.5 mg/ml of BSA, 0.2 mg/ml sonicated salmon sperm DNA for 1 h. The antibody–bead mixture was rotated overnight at 4 °C. Beads were transferred to 1.5-ml no stick tubes (Alpha Laboratories) and washed following the protocol described by Engelen et al. [19]. The final three washes were performed with 0.2 M HEPES pH 8.5 and samples eluted twice in 60 µl (each) of 50 mM HEPES pH 8.5, 5 mM dithiothreitol (DTT), 5% (weight per volume; w/v) SDS at 90 °C for 5 min on a thermocycler with shaking (800 rpm). A small portion of the IP eluate (10%) was run on a polyacrylamide gel and stained with Silver staining to check the quality of the ChIP and reverse crosslinking. The remaining material was treated with 20 mM chloroacetamide for 30 min at room temperature. Excess chloroacetamide was quenched through the addition of DTT to a final concentration of 40 mM. Eluted proteins were then processed using a modified version of the SP3 protocol [20, 21]. Specifically, 250 µg of a combined and rinsed stock of Sera-Mag carboxylate-modified magnetic beads was combined with eluted proteins in a working volume of 100 µL. Aggregation of proteins to the surface of the magnetic beads was driven via addition of acetonitrile to a final proportion of 80% (volume per volume; v/v). SP3 reactions were incubated for 5 min at room temperature, placed on a magnetic rack, and the supernatant was discarded. The beads were reconstituted in 800 µL of 80% (v/v) ethanol, and placed on the magnetic rack again for supernatant removal. After supernatant removal, beads were reconstituted in 100 mM HEPES pH 7.3 containing trypsin + rLysC (Promega) at a ratio of approximately 1:100 µg (trypsin:sample) and incubated at 37 °C for 18 h in a ThermoMixer with shaking at 1000 rpm. Peptide-containing supernatants were recovered via centrifugation at 12,000* g* for 2 min along with use of a magnetic rack and then desalted using C18 SlitPlates (Glygen Scientific). Desalting was carried out by activating the column with methanol, rinsing with 0.1% (v/v) trifluoroacetic acid, sample binding, rinsing with 4% (v/v) methanol in 0.1% (v/v) formic acid, and final elution in 60% (v/v) methanol in 0.1% (v/v) formic acid. Desalted peptides were dried in a SpeedVac and reconstituted in a solution of 1% (v/v) dimethylsulfoxide (DMSO) and 1% (v/v) formic acid.

#### MS analysis

DDA-MS analysis was carried out on an Orbitrap Fusion instrument equipped with an EASY-nLC 1200 liquid chromatography (LC) system. For analysis, peptides were initially trapped on a pre-column (100 µm × 3 cm, 1.9 µm C18 Reprosil-Pur Basic beads from Dr. Maisch) and then separated by an analytical column (100 µm × 25 cm, 1.9 µm C18 Reprosil-Pur Basic beads) coupled to an in-house pulled tip electrospray emitter (~ 20 µm) with a 90-min gradient at a flow rate of 400 nL/min. Data acquisition on the Orbitrap Fusion used a standard data-dependent acquisition scheme. Specifically, the Orbitrap Fusion was globally set to use a positive ion spray voltage of 2200 V, an ion transfer tube temperature of 275 °C, a default charge state of 1, and an RF Lens setting of 60%. MS1 survey scans covered a mass range of 380–1500 m/z at a resolution of 120,000 with an automatic gain control (AGC) target of 2e5 and a max injection time of 30 ms. Precursors for tandem MS/MS (MS2) analysis were selected using monoisotopic precursor selection (“Peptide” mode), charge state filtering (2–4z), dynamic exclusion (20 ppm low and high, 30 s duration), and an intensity threshold of 5e3. MS2 scans were carried out in the ion trap using the “Rapid” scan mode, a fixed-first mass of 110 m/z, a higher-energy collision dissociation setting of 32%, an AGC target of 1e4, a max injection time of 30 ms, and an isolation window setting of 1 m/z. The total allowable MS2 cycle time was set to 4 s. MS1 data were acquired in profile mode, and MS2 in centroid. Resulting MS data were processed to obtain peptide and protein identifications using FragPipe (v17.1) with MSFragger (v3.4) [22] and Philosopher (v4.1.1) [23] with the default settings. Specifically, raw data were searched using MSFragger using the settings: (1) 20 ppm precursor error; (2) 0.6 Daltons fragment error; (3) 2 missed cleavages; (4) + 15.99@M, + 42.01@N-term, + 8.01@K, + 10.00@R as variable modifications; (5) + 57.02@C as a fixed modification; (6) Max precursor charge = 4. Data was searched against the Uniprot human database (version 2021–12) using a target-decoy strategy and filtered to provide an ~ 1% FDR. Quantification of light and heavy peptide peaks was performed using the IonQuant node within FragPipe with the default settings. Proteins with a positive log_2_ fold change (mean HEK-*KMT2D*^WT^ replicates / mean HEK-*KMT2D*^LOF^ replicates) and at least one unique peptide in a replicate were considered candidate KMT2D interactors.

### Identifying *KMT2D* mutations across cancer types

Mutation annotation format (MAF) file from TCGA pan-cancer dataset, representing 10,217 tumour samples across 33 cancer types [24], was downloaded from https://gdc.cancer.gov/about-data/publications/pancanatlas/ (mc3.v0.2.8.PUBLIC.maf; accessed on June 15th, 2022). Additionally, we downloaded mutation annotations from supplementary files from B-cell non-Hodgkin lymphoma (B-NHL) datasets (117 samples) [25–27], medulloblastoma (MED) dataset (53 samples) [28], and a small cell lung cancers (SCLC) dataset (110 samples) [29], and annotated LOF mutations (nonsense mutations, frameshift insertions and deletions, and nonstop mutations). We used TCGA study abbreviations, except for B-NHL, which includes diffuse large B-cell lymphoma, follicular lymphoma, mantle cell lymphoma, and nodal marginal zone lymphomas, and COAD/READ, which includes colon and rectal adenocarcinomas. The following are the cancer type abbreviations used: adrenocortical carcinoma (ACC), bladder urothelial carcinoma (BLCA), B-cell non-Hodkin lymphoma (B-NHL), breast invasive carcinoma (BRCA), breast fibroepithelial (BRFE), cervical squamous cell carcinoma and endocervical adenocarcinoma (CESC), cholangiocarcinoma (CHOL), colon and rectal adenocarcinoma (COAD/READ), oesophageal carcinoma (ESCA), glioblastoma multiforme (GBM), head and neck squamous cell carcinoma (HNSC), kidney chromophobe (KICH), kidney renal clear cell carcinoma (KIRC), kidney renal papillary cell carcinoma (KIRP), acute myeloid leukaemia (LAML), brain lower-grade glioma (LGG), liver hepatocellular carcinoma (LIHC), lung adenocarcinoma (LUAD), lung squamous cell carcinoma (LUSC), medulloblastoma (MED), mesothelioma (MESO), ovarian serous cystadenocarcinoma (OV), pancreatic adenocarcinoma (PAAD), pheochromocytoma and paraganglioma (PCPG), prostate adenocarcinoma (PRAD), sarcoma (SARC), small cell lung cancer (SCLC), skin cutaneous melanoma (SKCM), stomach adenocarcinoma (STAD), testicular germ cell tumours (TGCT), thyroid carcinoma (THCA), thymoma (THYM), uterine corpus endometrial carcinoma (UCEC), uterine carcinosarcoma (UCS), and uveal melanoma (UVM).

### In silico genetic network mapping

#### Essentiality network maps

Essentiality mapping and in silico genetic interaction screening were performed using our R software package GRETTA (v0.99.2) [30] with DepMap public release version 20Q1 [31]. Briefly, co-essentiality mapping uses fitness effect scores derived from DepMap’s whole-genome CRISPR-Cas9 knockout screens, which targeted 18,333 genes in each of the 739 cancer cell lines. GRETTA calculated and ranked Pearson correlation coefficients between fitness scores of *KMT2D* and each screened gene. For each targeted gene in the genome-wide CRISPR KO screen, cell lines with missing gene effect scores were removed from the analysis in order to have a complete data set for Pearson correlation analysis. This resulted in 17,599 and 734 correlation coefficients calculated from 724 and 681 cancer cell lines, respectively. We corrected the *p*-values for multiple testing using permutation tests, which randomly sampled gene effect scores 100,000 times. A gene pair was considered co-essential if its Pearson correlation permutation-adjusted *p*-value was < 0.05 and its coefficient was greater than the inflection point of the positive curve, and anti-essential if its *p*-value was < 0.05 and less than the inflection point of the negative curve.

#### Identifying DepMap cell lines

Using GRETTA (0.99.2) [30], we queried *KMT2D* and *WRN* mutations from a total of 739 cancer cell lines in DepMap. These lines have WGS or whole-exome sequencing (WES) and RNA sequencing data, in addition to, genome-wide CRISPR-Cas9 KO screens [32, 33]. Whole proteome quantification and global histone quantification were only available for a subset of 375 and 897 cancer cell lines, respectively [32, 34]. The microsatellite status of DepMap cell lines was identified from supplementary files provided by Ghandi et al. [32]. As performed previously [6, 30], we used GRETTA to query the genomic data (MAF and copy number data files) to identify *KMT2D*^LOF^ and *WRN*^LOF^ mutant cancer cell lines (homozygous, trans-heterozygous, and heterozygous LOF mutants) and *KMT2D*^WT^ and *WRN*^WT^ control cell lines. Only trans-heterozygous lines (lines with more than one LOF mutation or a combination of LOF mutation and copy number loss) and heterozygous lines were available for the *KMT2D* pan-cancer group. Since trans-heterozygous LOF mutant groups are more likely to be KMT2D deficient than heterozygous lines [35], we only used these lines in the *KMT2D*^LOF^ cell line group (16 lines). All LOF mutants (trans-heterozygous and heterozygous *KMT2D*^LOF^ lines) lines were considered in the *KMT2D* context-specific screens. For the pan-cancer *WRN* genetic screen, only heterozygous LOF mutants (176 lines) were available. To match DepMap cell lines to the appropriate cancer types, we used the “disease” and “disease subtype” columns provided in the cell line annotation file (sample_info.csv [32]) according to Additional file 1: Table S1. The normalised *KMT2D* TPM gene expression and protein expression values were extracted using GRETTA.

#### Differential lethality analysis

As described previously [6, 30], we used GRETTA to perform pairwise Mann–Whitney *U* tests comparing lethality probabilities for all 18,333 genes targeted in DepMap’s CRISPR-Cas9 KO screen between LOF mutant lines and WT lines to obtain *p*-values. *P*-values were adjusted for multiple testing using a permutation approach by randomly resampling 10,000 of the lethality probability scores. We used a threshold of adjusted *p*-value < 0.05 to identify candidate GIs of *KMT2D* in each screen, which were then prioritised (described in detail below). For visualisation, we calculated the genetic interaction score:

$$Genetic\;interaction\;score=-{log}_{10}\lbrack adjusted\;p\;value\rbrack\;x\;{log}_2\lbrack\frac{median\;LOF\;group\;lethality\;probability}{median\;WT\;group\;lethality\;probability}\rbrack$$

#### Prioritising candidate GIs

Candidate *KMT2D* interactors were prioritised according to their GI significance and their drug tractability, similar to the approaches used by Behan et al. [8]. Firstly, candidates were grouped into three tiers (tiers I, II, and III) based on their GI significance. Tier I candidates were the most significant group with adjusted Mann Whitney *U* test *p*-values < 0.01, an absolute log_2_ fold change > 2 between median lethality probabilities (LOF group / WT group), and a minimum lethality probability of 0.5 in at least one group (WT or LOF). The following GI tiers II and III used progressively less stringent thresholds.

Next, candidates were grouped based on drug tractability (groups I, II, and III). We used the Open Target Platform [36], a database that contains a collection of tractability assessments based on sources, such as UniProt, HPA, PDBe, DrugEBIlity, ChEMBL, Pfam, InterPro, Complex Portal, DrugBank, Gene Ontology, and BioModels (https://platform-docs.opentargets.org/target/tractability/; tractability_v23-02.tsv; accessed on February 15th, 2022), which is a strategy developed by Brown et al. [37] and Schneider et al. [38]. Using this Open Target, we assessed whether the candidate GI encodes a protein for which there is already a known drug or, based on its protein structure, the likelihood of a drug being developed. Drug tractability group I contains GI candidates that are most tractable and encodes proteins that are currently targetable using approved or phase I, II, and II small molecule inhibitors or antibodies (buckets 1–3; documented on https://github.com/chembl/tractability_pipeline_v2/tree/master/; accessed on accessed on February 15th, 2022). Group II contains targets that have high-quality protein structure, ligand, and pocket annotations and cellular location annotation (buckets 4–6). Group III contains targets with proteins that have medium to low-quality pocket annotation, cellular location annotation, or are known members of the druggable protein family (buckets 7–9).

Finally, GI candidates were prioritised by combining the GI significance and drug tractability assessments (class A, B, C, and D). With progressively less likelihood of targetable GIs, class A contained GIs with the highest significance with targetable proteins, and class D containing GI with the least significance with no evidence of tractability.

### TCGA cohort analyses

#### Identifying *KMT2D* mutations

We used cBioPortal [39, 40] to download clinical (including MSIsensor, MANTIS, and TMB scores) and genomic data from TCGA Pan-Cancer Atlas Studies (33 cancer types). A TCGA case was considered to have MSI if the MSIsensor and MANTIS scores were ≥ 10 and ≥ 0.4, respectively, as previously determined [41, 42]. We annotated cases with *KMT2D* LOF alterations as having nonsense mutation, frameshift insertions, frameshift deletions, or deep copy number loss. Cases with missense or silent mutations were excluded.

#### *KMT2D* and *TP53* oncoprint in COAD/READ cases

Oncoprint showing *KMT2D* and *TP53* mutations in 565 TCGA-COAD/READ cases was generated using cBioPortal [39, 40].

#### Mutational signature analysis

A matrix of 96 trinucleotide substitutions were downloaded from Alexandrov et al. [43] at https://www.synapse.org/#!Synapse:syn11726618/. The matrix was analysed in Python (v3.9) using the SigProfilerAssignment [44] library to score single base pair substitution (SBS) signature activity scores with COSMIC (v.3.3), exome normalisation, and default settings.

#### Neoantigen and HRD scores and RNA-seq data

Neoantigen scores and HRD scores were downloaded from Thorsson et al. [45] and RNA-seq count data were downloaded from UCSC Xena Hub [46]. Additional immune marker analyses are described below.

### POG cohort analyses

#### Ethics approval, consent to participate, and enrollment criteria

The main objectives for our POG cohort analyses in this manuscript were to retrospectively validate TCGA cohort findings using whole-genome and whole-transcriptome analysis. Our work, involving POG program patients, was approved by the University of British Columbia–BC Cancer Research Ethics Board (H12-00137, H14-00681). The POG program (registered under clinical trial number NCT02155621) is an ongoing single-arm study conducted at BC Cancer with the primary objective of collecting blood and cancer biopsies from patients for genomic and transcriptomic sequencing and analysis [47]. The primary outcomes are to assess the influence of genomic data on clinical decision-making and to generate a catalogue of cancer genomes, as previously described in Pleasance et al. [15] [48]. All patients analysed in this study gave informed written consent and were enrolled into POG as described previously [14, 15, 48]. The first (POG01) and last patient (POG1448) in this study provided their consent on July 7th, 2012, and January 13th, 2023, respectively.

#### Tissue collection

Tissue collection and sequencing were performed as in Pleasance et al. [48] for 820 patient samples. Tumour specimens were collected using needle core biopsies or tissue resection, snap frozen generally in optimal cutting temperature compound, reviewed by pathologists, and DNA and RNA libraries constructed and sequenced. Genome data were used to detect somatic alterations, including SNVs, copy number variants, loss of heterozygosity, and structural variants (such as gene fusions).

#### Whole-genome and transcriptome libraries and sequencing

PCR-free genomic DNA libraries and poly-A selected RNA libraries were constructed and sequenced, and reads were aligned to the GRCh38 genome reference. Tumour genomes were sequenced to a target depth of 80X coverage and normal peripheral blood samples to a target depth of 40X coverage on Illumina HiSeq instruments. Details regarding library preparation, sequencing, and quality control can be found in Pleasance et al. [48]. In cases with RNA integrity numbers < 7.0, ribosomal RNA depletion RNA sequencing was employed as described in Pleasance et al. [48]. Recent samples (since POG1046) were processed using an updated ribosomal RNA depletion RNA sequencing protocol, which is described below.

To remove cytoplasmic rRNA and mitochondrial ribosomal RNA (rRNA) species from total RNA, NEBNext rRNA Depletion Kit for Human/Mouse/Rat was used (NEB, E6310X). Enzymatic reactions were set-up in a 96-well plate (Thermo Fisher Scientific) on a Microlab NIMBUS liquid handler (Hamilton Robotics, USA). One hundred fifty nanograms of DNase treated total RNA in 6 µL was hybridised to rRNA probes in a 8.5 µL reaction. Heat-sealed plates were incubated at 95 °C for 2 min followed by incremental reduction in temperature by 0.1 °C per second to 22 °C (730 cycles). The rRNA in DNA hybrids were digested using RNase H in a 11 µL reaction incubated in a thermocycler at 50 °C for 30 min. To remove excess rRNA probes (DNA) and residual genomic DNA contamination, DNase I was added in a total reaction volume of 26 µL and incubated at 37 °C for 30 min. RNA was purified using RNA MagClean DX beads (Aline Biosciences, USA) with 15 min of binding time, 7 min clearing on a magnet followed by two 70% ethanol washes, 5 min to air dry the RNA pellet and elution in 18 µL DEPC water. The plate containing RNA was stored at − 80 °C prior to cDNA synthesis.

First-strand cDNA was synthesised from the purified RNA (minus rRNA) using the Maxima H Minus First Strand cDNA Synthesis kit (Thermo-Fisher, USA) and random hexamer primers at a concentration of 8 ng/µL along with a final concentration of 0.04 µg/µL Actinomycin D, followed by PCR Clean DX bead purification on a Microlab NIMBUS robot (Hamilton Robotics, USA). The second-strand cDNA was synthesised following the NEBNext Ultra Directional Second Strand cDNA Synthesis protocol (NEB) that incorporates dUTP in the dNTP mix, allowing the second strand to be digested using USER™ enzyme (NEB) in the post-adapter ligation PCR and thus achieving strand specificity.

cDNA was fragmented by Covaris LE220 sonication for 130 s (2 × 65 s) at a “Duty cycle” of 30%, 450 Peak Incident Power (W), and 200 Cycles per Burst in a 96-well microTUBE Plate (P/N: 520,078) to achieve 200–250 bp average fragment lengths. The paired-end sequencing library was prepared following Canada’s Micheal Smith Genome Sciences Centre strand-specific, plate-based library construction protocol on a Microlab NIMBUS robot (Hamilton Robotics, USA). Briefly, the sheared cDNA was subject to end-repair and phosphorylation in a single reaction using an enzyme premix (NEB) containing T4 DNA polymerase, Klenow DNA Polymerase, and T4 polynucleotide kinase, incubated at 20 °C for 30 min. Repaired cDNA was purified in 96-well format using PCR Clean DX beads (Aline Biosciences, USA), and 3’ A-tailed (adenylation) using Klenow fragment (3’ to 5’ exo minus) and incubation at 37 °C for 30 min prior to enzyme heat inactivation. Ligation was done using TruSeq adapters at 20 °C for 15 min. The adapter-ligated products were purified using PCR Clean DX beads followed by indexed PCR using NEBNext Ultra II Q5 Master Mix (NEB) with USER enzyme (1 U/µL, NEB) and dual indexed primer set. PCR parameters: 37 °C for 15 min, 98 °C for 1 min followed by 13 cycles of 98 °C 15 s, 65 °C 30 s and 72 °C 30 s, and then 72 °C 5 min. The PCR products were purified and size-selected using a 1:1 PCR Clean DX beads-to-sample ratio (twice), and the eluted DNA quality was assessed with Caliper LabChip GX for DNA samples using the High Sensitivity Assay (PerkinElmer, Inc. USA) and quantified using a Quant-iT dsDNA High Sensitivity Assay Kit on a Qubit fluorometer (Invitrogen) prior to library pooling and size-corrected final molar concentration calculation for Illumina sequencing with paired-end 150 base reads.

#### Variant calling and *KMT2D* mutation selection

Somatic point mutations and small insertions and deletions are identified using Strelka 2.6.2 [49] and Mutect2 from GATK 4.0.10.0 [50]. Variants from these tools are intersected using RTGTools [51] to generate the calls as previously described [52]. We defined POG cases with *KMT2D* LOF alterations as those having somatic nonsense mutations, frameshift insertions, frameshift deletions, or deep copy number loss indicating likely homozygous deletions. Cases with missense or silent mutations were not considered and were excluded from the analysis entirely. POG cases with WT *KMT2D* alleles were defined as those with no somatic alteration and neutral copy number. Germline alterations were not considered in this study.

#### Immune marker analysis

Tumour mutation burden (TMB, mutations per megabase) is calculated by adding small mutations and small indels within the tumour sample generated in the above [52] and dividing by the effective genome size (2934.876451 Mb). MSI were detected using genome data according to Pleasance et al. [48] and HRDetect scores were calculated using a logistic regression model [53]. The model uses six mutation signatures associated with HRD as inputs into the algorithm: (i) Signature (SBS) 3, (ii) Signature (SBS) 8, (iii) SV signature 3, (iv) SV signature 5, (v) the HRD index, and (vi) the fraction of deletions with microhomology. All signatures were normalised and log transformed as previously described [48]. Additional immune marker analyses are described below.

### MSK-IMPACT cohort analyses

Targeted panel gene sequencing, survival, and TMB data from the MSK-IMPACT cohort were downloaded from cBioPortal (https://cbioportal-datahub.s3.amazonaws.com/tmb_mskcc_2018.tar.gz; accessed March 15th, 2022; [13]). The MSK-IMPACT assay identifies somatic exonic mutations in 468 cancer-associated genes using both tumour-derived and matched germline normal DNA [13]. We identified cases with *KMT2D*^LOF^ alterations, when a case harboured either a frameshifting INDEL or a nonsense deleterious mutation. We inferred a case was MSI when a LOF alteration(s) (i.e., frameshift INDELs and nonsense mutations) in one of the MMR genes (*MLH1*, *MLH3*, *PMS2*, *MSH2*, *MSH3*, and *MSH6*) was observed. TMB was calculated as per Samstein et al. [13] as the total number of somatic mutations normalised to the exonic coverage of the MSK-IMPACT panel in megabases.

### Survival analyses

Survival analyses were performed using R package ggsurvfit (v0.2.1). Differences in nonparametric survival functions were assessed across groups using log-rank tests with the ggsurvfit package. Groups with less than 10 cases or without enough observations to determine 50% survival probability were not analysed.

### Additional immune marker analyses

Cytolytic activity scores were calculated as the geometric mean of *GZMA* and *PRF1* expression (FPKM), as performed by Rooney et al. [54]. M1/M2 macrophage scores were calculated according to Pender et al. [55], as the mean expression (FPKM) of *CXCL11*, *IDO1*, *CCL19*, *CXCL9*, *PLA1A*, *LAMP3*, *CCR7*, *APOL6*, *CXCL10*, and *TNIP3*. IFNγ signature scores were calculated according to Ayers et al. [56] by calculating the mean expression from ten genes associated with IFNγ signalling (*CCR5*, *CXCL10*, *CXCL11*, *CXCL9*, *GZMA*, *HLA-DRA, IDO1*, *IFNG, PRF1*, and *STAT1*). Immune cell infiltration proportion and absolute counts were calculated using the Cibersortx webportal (https://cibersortx.stanford.edu/) with default settings and the LM22 signature matrix file [57]. Immune checkpoint response scores were calculated using the PredictIO webportal (https://predictio.ca/), which uses a gene signature-based method to predict responders to ICI treatment [58].

### Enrichment analysis and gene function annotation

We used clusterProfiler (v3.16.1) [59] to identify pathway and protein complexes significantly enriched (q-value < 0.05) in the *KMT2D* essentiality network and protein interaction network, as well as to annotate functions of gene in the GI networks (unadjusted *p*-value < 0.05). Given many related terms with similar sets of genes associated with them, we grouped the terms based on their degree of similarity calculated with the Jaccard index, as performed in Takemon et al. [6]. We performed hierarchical clustering of the Jaccard index between pairs and the number of distinct clusters was determined using the gap statistic, which calculated the optimal number of clusters (up to 15 clusters) by iteratively bootstrapping 1000 times using the cluster (v2.1.4) package.

### Analysis of *WRN* KO dependency and WRN inhibitor sensitivity validated cell lines

Cancer cell lines that were validated to be *WRN* KO-(in)dependent by Behan et al. [8], Chan et al. [60], Kategaya et al. [61], Lieb et al. [62], and Picco et al. [63] and validated to be (in)sensitive to WRN inhibitors (HRO761 and VVD-133214) by Ferretti et al. [16] and Baltgalvis et al. [17], were identified and cross-referenced to DepMap cell lines with WGS/WES annotation (20Q1 and 23Q4). We note that the WRN inhibitor insensitive cell line HCT8, tested by Baltgalvis et al. [17], is identical to HRT18 (https://www.atcc.org/products/ccl-244). For each cell line, we annotated with the reported *WRN* KO or WRN inhibitor dependence and microsatellite status. Cell lines validated by Ferretti et al. [16] were annotated as WRN inhibitor sensitive or insensitive, when the cell line required low or high WRN inhibitor concentrations, respectively, to achieve 50% growth inhibition in the colony-formation assay. Using DepMap WGS/WES annotations, we identified and annotated LOF alterations, including copy number loss, nonsense mutations, frameshift insertions and deletions, and nonstop mutations, affecting *KMT2D* and MMR genes (namely *EXO1*, *MBD4*, *MLH1*, *MSH2*, *MSH3*, *MSH5*, *MSH6*, *PMS1*, *PSM2*, and *RFC1*). Fisher’s exact tests were performed to determine co-occurrence between *KMT2D*/MMR gene LOF mutations and WRN dependence, and between *KMT2D* and MMR gene LOF alterations.

### Microsatellite repeat expansion analysis

We identified MSI DepMap cancer cell lines with WGS data that carried a WT *KMT2D* allele or LOF alterations (WT line: SKMEL2 and LOF lines: CCK81, LOVO, SW48, LS180, MFE319, and RKO) as well as two MSS lines (NCIH747 and COLO201; SRA project ID: PRJNA523380; SRA run IDs in Additional file 2: Table S2A). We downloaded the raw.fastq data using SRA-Toolkit (v2.11.3; https://trace.ncbi.nlm.nih.gov/Traces/sra/sra.cgi?view=software/) and aligned to GRCh38 using bwa (v0.7.17) [64]. We used default ExpansionHunter Denovo (v0.9.0) [65] settings to detect AT-motifs and filtered for regions that contained at least two in-repeat reads (i.e. one read pair maps within and the other outside of a repeat region).

### Statistical analyses

All statistical analyses were conducted using R statistical software (v4.2.2) [66] unless otherwise stated.

## Results

### *KMT2D* essentiality network includes genes associated with histone modification, transcription, mitotic cell cycle regulation, glycolysis, and DNA replication

**Fig. 1:**
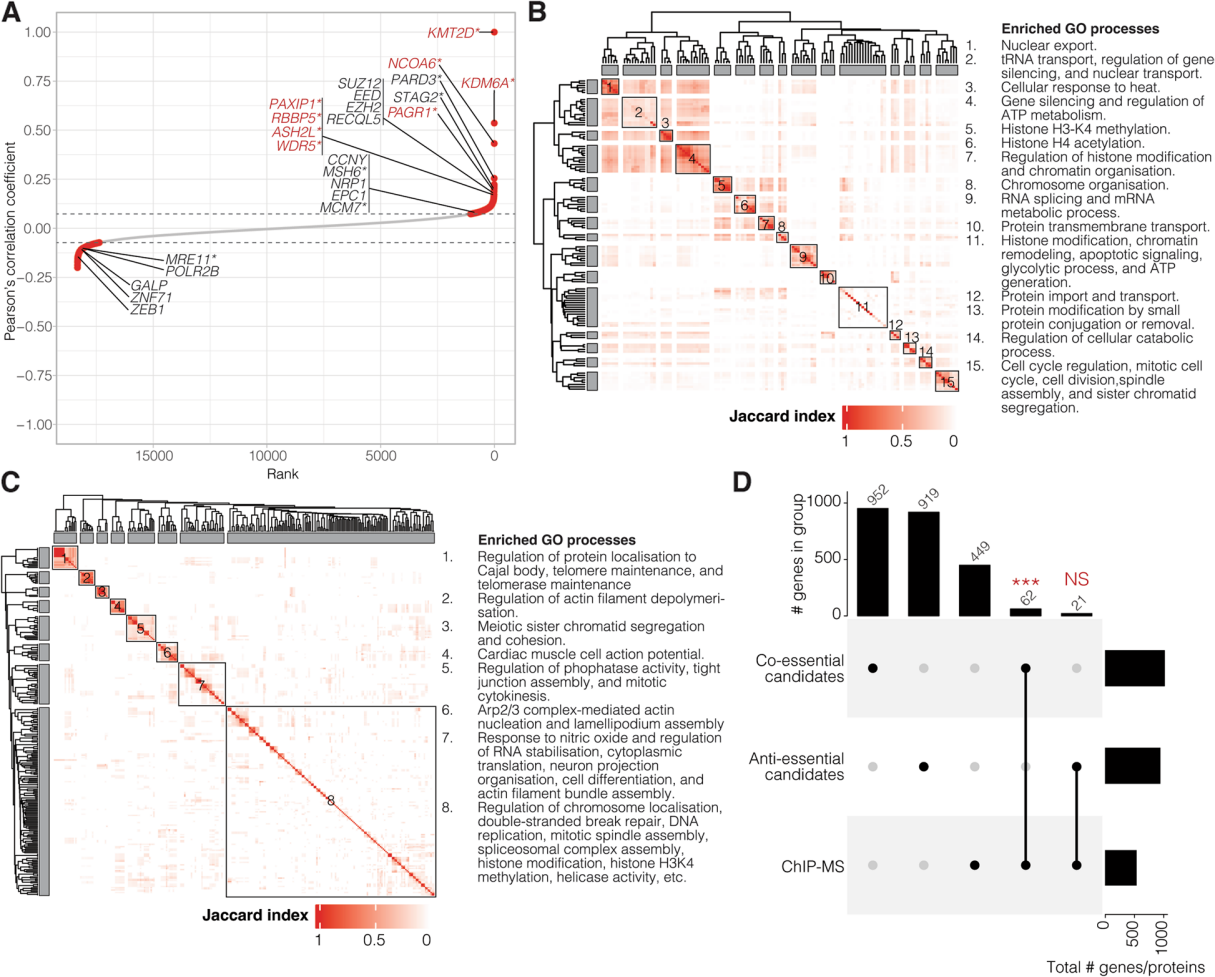
H3K4 methylation, chromatin segregation, and spindle assembly are enriched in *KMT2D* essentiality and protein networks. **A** Ranked Pearson’s correlation coefficient between *KMT2D* and 18,333 genes. The dotted line denotes the inflection point of the positive and negative curves. Red points indicate candidate co-essential (positive coefficient scores) and anti-essential (negative coefficient scores) genes. Genes labelled in red indicate COMPASS complex members. * indicate KMT2D protein interactors detected in ChIP-MS assay in (C-D). **B-C** Heatmap showing Jaccard index similarities between 135 significantly enriched GO biological processes from *KMT2D *co-essential candidate genes (B) and 268 significantly enriched GO terms from KMT2D ChIP-MS (C). Jaccard index-based hierarchical clustering was used to group the GO terms into distinct functions (left), and the functions are summarised in the text (right). **D** Upset plots showing the overlap of genes/proteins detected in KMT2D ChIP-MS and the essentiality network mapping. Bars on the right show the total number of candidates in each row. Bars on the top denote the unique (single black dot) or overlapping (two connected black dots) genes/proteins detected between groups. Fisher’s exact test was to determine significant overlaps: *p*-value *** < 0.001 and not significant (NS) > 0.05

To map *KMT2D*’s essentiality network, we used GRETTA [30, 67], a software approach that we developed to leverage the public cancer dependency map (DepMap) data platform to map genetic networks (gene essentiality networks and genetic interaction networks). GRETTA uses genomic and transcriptomic data and CRISPR perturbation screening data generated by DepMap from cancer cell lines to map genetic networks in silico [30]. Similar approaches have been used to map genetic networks of cancer-associated genes [3, 5, 8]. Using the essentiality network mapping functions in GRETTA, we compared the fitness effects of *KMT2D* knockout (KO) to the effects of knocking out 18,333 genes that were screened by DepMap in 739 cancer cell lines across > 30 cancer types (Methods; [31]). We identified 1014 candidate co-essential genes and 940 candidate anti-essential genes in *KMT2D*’s essentiality network (Fig. 1A; Additional file 3: Table S3A). Several co-essential and anti-essential candidates have been previously linked to *KMT2D* or its known functions, and we provide a summary of their KMT2D-associated functions in Table 1. Notably, we did not observe *KMT2C* as a co-essential gene, indicating that KMT2C and KMT2D may be functionally distinct, which supports a previous observation that identified independent functions of these genes [68]. These candidate genes confirm that essentiality networks can capture known protein–protein interactions and functionally related genes.

**Table 1:** Candidate co-/anti-essential genes and protein interactors with known relations to KMT2D membership/function(s)

Candidates	Candidate membership/function(s)	Known KMT2D membership/function(s)
ASH2L*, RBBP5*, WDR5*, KDM6A*, NCOA*, and PAGR1, PAXIP1* (co-essential)	Core and KMT2D-specific COMPASS protein complex members [69–71]	COMPASS protein complex member [80]
*CCNY, EPC1*, *NRP1*, and PARD3* (co-essential) *GALP*, *ZEB1*, and *ZNF71* (anti-essential) KIF5B and RAP1A (ChIP-MS)	Mutations associated with Kabuki and Kabuki-like syndromes [81]	Loss known to be associated with Kabuki syndrome [82]
*EED*, *EZH2*, and *SUZ12* (co-essential)	Polycomb repressive complex 2 members with roles depositing H3K27me2/3 repressive marks [83]	Deposits H3K4me3 marks that are known to inhibit PRC2 H3K27me2/3 activity [84–86]
*RECQL5* (co-essential)	Known interactor of KMT2D [72] RecQ DNA helicase with roles in double-stranded DNA break repair, transcription stress, and genomic instability [76, 87]	Known interactor of RECQL5 [72] Associated with transcription stress, and genomic instability [87]
MRE11* (anti-essential)	MRN complex member responsible for recognising, repairing, and signalling of double-stranded breaks [88]	Loss of KMT2D leads to reduced recruitment of MRE11 to replication forks [89]
*POLR2B* (anti-essential) POLR2A (ChIP-MS)	KMT2D interactor encoding RNA polymerase II [72]	RNA polymerase II interactor. Loss of KMT2D affects POLR2A-associated histone methylation [72]
p53 (ChIP-MS)	Responds to stress stimuli and has mutation-dependent tumour suppressive and oncogenic roles [90]	p53 and mutant p53 interact with KMT2D [91, 92]

*Indicates genes whose encoded protein was also detected in the KMT2D ChIP-MS analysis

Next, we performed an enrichment analysis to determine whether there were over-represented biological processes among the candidate co-/anti-essential genes. We identified 135 and eight gene ontology (GO) terms that were significantly enriched in our list of candidate co-essential and anti-essential genes, respectively (q-value < 0.05; Additional file 3: Table S3B; Methods). We summarised the GO terms enriched in co-essential candidates into 15 distinct groups based on similarities in genes associated with the terms (Fig. 1B; Additional file 3: Table S3C; Methods). We note that group 11 contained candidate co-essential genes that were significantly enriched for roles that included histone modification, chromatin remodeling, and glycolytic processes, and their low jaccard index scores indicated that these enriched co-essential gene sets were unique to these GO terms. As expected for *KMT2D*, terms associated with histone modification were enriched. Terms related to cell cycle, cell division, spindle assembly, and sister chromatid segregation were also enriched, including novel candidate co-essential genes and the known *KMT2D*-associated genes *PAGR1*, *PAXIP1* [69–71], *RECQL5* [72], *INO80* [73], and *STAG2* [74]. Notably, these genes also participate in DNA damage repair [75–78]. We also found statistically significantly enriched terms associated with glycolytic processes and ATP generation, which included novel candidate co-essential genes. Among the anti-essential genes, we identified eight significantly enriched GO terms—including DNA-templated transcription initiation, transcription initiation from the RNA polymerase II promoter, histone H3 acetylation, positive regulation of DNA replication, telomere maintenance, telomere organisation, and regulation of telomere maintenance (q-value < 0.05; Additional file 3: Table S3B; Methods). In addition to KMT2D’s known role in histone modification and transcription initiation [79], our results indicate that its role may extend into the regulation of cell cycle, cell division, telomere maintenance, glycolysis, and DNA replication.

### KMT2D’s protein interaction network is significantly enriched for functions in histone methylation, chromosome segregation, and DNA replication and repair

To determine whether *KMT2D*’s candidate co-/anti-essential genes also shared physical protein–protein interactions, we performed KMT2D chromatin immunoprecipitation followed by tandem mass spectrometry (ChIP-MS) in human embryonic kidney (HEK293A) cell lines. Given KMT2D’s role as a histone modifier, we chose to capture protein interactions using ChIP to identify chromatin-specific interactions rather than using the common IP-MS techniques, which capture general cellular protein interactions. We also used an experimentally tractable cell line, HEK293A, which made it feasible for us to perform ChIP-MS assays and generate *KMT2D* KO controls in this cellular background (Methods). Furthermore, since *KMT2D* alterations are frequently found across cancer types [12], compatible with the notion that KMT2D plays a role(s) relevant to a range of cancer biologies, we rationalised that ChIP-MS in HEK293A cell lines could capture KMT2D protein interactions that may exist across several cancer types.

Using this method, we identified 532 candidate KMT2D chromatin interactors, which included 14 proteins that were previously described to interact or have functions that are related to those known to KMT2D (summarised in Table 1; Additional file 4: Table S4A; Methods; [93]). To highlight functions that were significantly enriched in proteins detected using KMT2D ChIP-MS, we performed an enrichment analysis and found 268 GO terms were significantly enriched in candidate KMT2D chromatin interactors (q-value < 0.05; Fig. 1C; Methods; Additional file 4: Table S4B). We summarised these terms into eight distinct groups and identified significant enrichment for proteins associated with the regulation of telomere maintenance, actin filament depolymerisation, sister chromatid segregation and cohesion, DNA replication, and double-stranded break repair (Fig. 1C; Additional file 4: Table S4C; Methods). We note that group 8 contained candidate protein interactors that were significantly enriched for roles that included DNA replication, double-stranded break repair, DNA replication, mitotic simple assembly, and histone H3K4 methylation, and their low Jaccard index scores indicated that these enriched sets of protein interators were unique to these GO terms. Additionally, similar to *KMT2D*’s co-essential candidates, protein interactors were also significantly enriched for functions in H3K4 methylation, chromosome segregation, and spindle assembly. Further highlighting their similarities, we found statistically significant overlap between KMT2D protein interactor candidates and co-essential gene candidates (62 proteins/genes overlapped; Fisher’s exact test *p*-value = 2.18 × 10^−8^; Fig. 1D; Additional file 4: Table S4D). However, the overlap between candidate protein interactors and anti-essential gene candidates was not significant (21 proteins/genes overlapped; Fisher’s exact test *p*-value = 0.12; Fig. 1D). Interestingly, among genes that shared both protein interaction and essentiality with *KMT2D*, we found *OGT* as a co-essential candidate. *OGT* encodes the nutrient-sensing enzyme O-GlcNAc transferase, which has been shown to modify SET1-COMPASS activities in *Drosophila* and interact with SET1A-COMPASS to promote depositions of H3k4me3 marks in mice [94]. OGT has also been implicated in the regulation of DNA replication and genomic integrity maintenance [95, 96]. However, OGT has not yet been directly associated with KMT2D. Notably, we also found that KMT2D shared protein interaction and gene essentiality with several proteins involved in DNA replication and repair, namely MCM7, MRE11, MSH6, and STAG2. MCM7 and MRE11 have been implicated in DNA replication-coupled repair [97], MSH6 participates in mismatch repair [98], and STAG2 is a member of the cohesin complex, responsible for the cohesion of sister chromatids, DNA replication fork progression, and maintaining genome stability [78, 99]. Gene functions have been inferred from their co-essential genes and protein interactors [5, 100]. The significant enrichment of proteins/genes involved in chromosome segregation and DNA replication in two independent networks (*KMT2D* essentiality and KMT2D protein interaction networks) supports the notion that KMT2D may also be involved in these functions, consistent with previously published data [72, 87] showing KMT2D’s role in genome integrity maintenance in addition to its canonical role in histone methylation.

### In silico genetic screens reveal *pan*-*cancer* and context-specific *KMT2D* GIs associated with mitotic processes, DNA repair, metabolism, and immune response

**Fig. 2:**
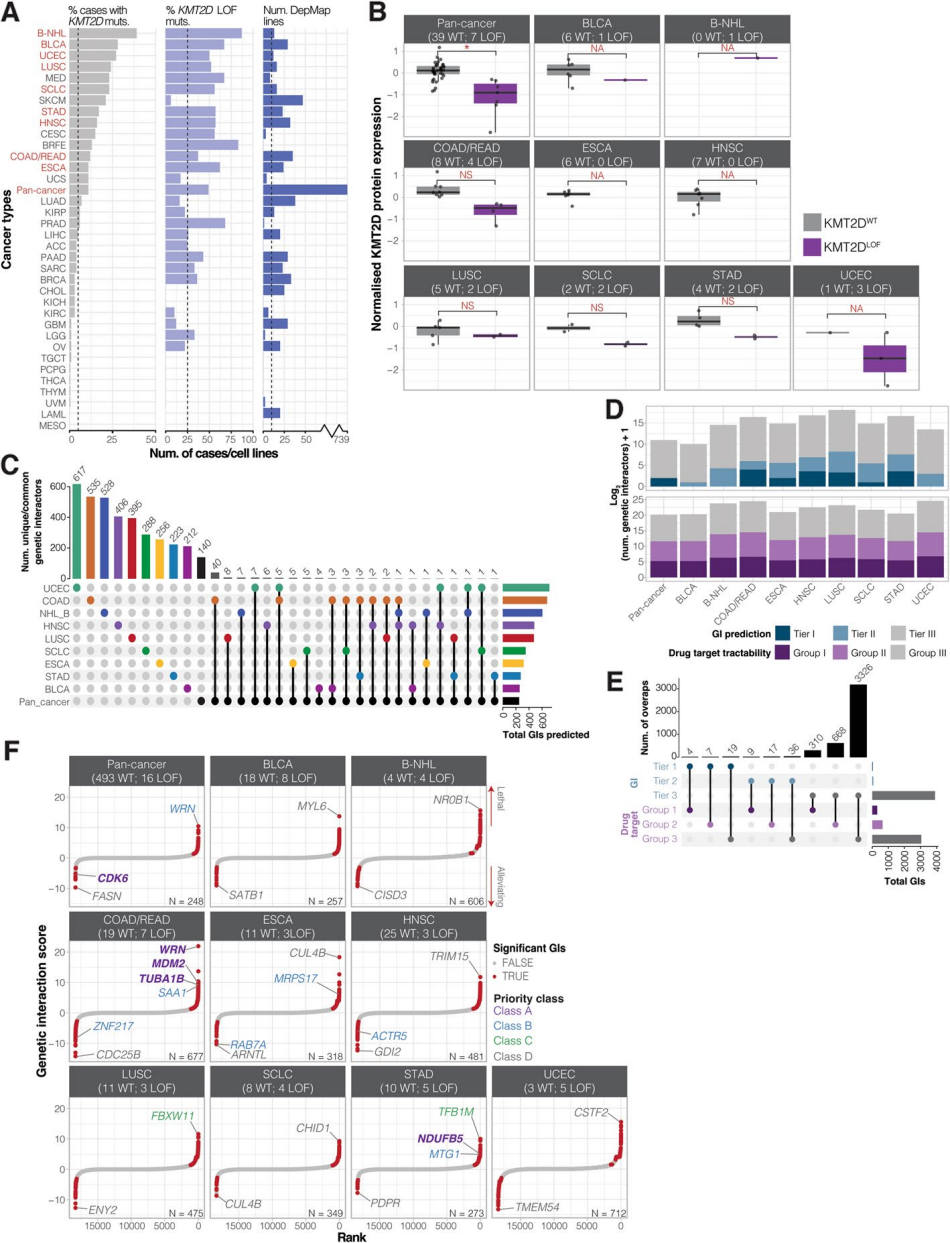
Identifying pan-cancer and cancer type-specific *KMT2D *GI networks. **A **(Left) Percentage of *KMT2D* small nucleotide mutations (SNVs) detected across 35 cancer types and a pan-cancer dataset. (Middle) Percentage of SNVs that are LOF mutations. (Right) The number of DepMap cancer cell lines for each category. Dotted lines show the threshold for each panel. Red labels indicate the ten datasets that passed all three thresholds and were selected for *in silico* screening. See Methods for cancer type abbreviations. **B** Normalised KMT2D protein expression for *KMT2D*^WT^ and *KMT2D*^LOF^ DepMap cancer cell lines. **C** Upset plot showing the number of unique or overlapping candidate GIs between screens. Bars on the right indicate the total number of GIs predicted in each screen. Bars on top indicate the number of GIs that are unique to or shared between the screens. Single coloured dots indicate unique GIs, while linked coloured dots indicate those that are shared between screens. **D** Bar plots showing the log_2_-transformed number of GI candidates + 1 grouped into GI tiers (top) and drug tractability groups (bottom). **E** Upset plot showing the number of GI candidates with overlapping priority groups. Right bars indicate the total number of candidates in a GI tier or tractability group. Top bars indicate the number of candidates shared between GI tiers and tractability groups indicated by linked coloured dots. **F** Ranked GI score summary for each of the ten *in silico* screens. (Bottom right corners) The total numbers of candidates. Class A, B, and the candidate with the highest and lowest GI scores are labelled (see Additional file 6: Table S5D)

We next used GRETTA [30] to perform pan-cancer and cancer type-specific in silico genome-wide KO screens, with the aim of identifying SL and AL interactors of *KMT2D*. To determine the cancer types that were most relevant to *KMT2D*^LOF^ cancers, we queried the datasets from The Cancer Genome Atlas (TCGA; 10,217 tumour samples across 33 cancer types [24]) and included additional datasets from cancer types that are known to frequently harbour *KMT2D* alterations (see Methods for details). We selected 14 cancer types in which at least 10% of samples harboured *KMT2D* mutations and where 25% of the total *KMT2D* mutations were LOF (Fig. 2A; Methods). Next, we selected cancer types with at least ten DepMap cancer cell lines, using the criterion of Behan et al. [8]. This resulted in the identification of nine cancer types (namely bladder urothelial carcinomas [BLCA], B-cell non-Hodgkin lymphoma [B-NHL], colorectal adenocarcinomas [COAD/READ], oesophageal carcinomas [ESCA], head and neck squamous cell carcinomas [HNSC], lung squamous cell carcinomas [LUSC], small cell lung cancers [SCLC], stomach adenocarcinomas [STAD], and uterine corpus endometrial carcinoma [UCEC]), in which cancer type-specific in silico GI screen was performed (Fig. 2A; Methods). In addition to these nine, we also performed a pan-cancer screen that included all DepMap cell lines. Given that the pan-cancer screen would include cell lines consisting of various cancer types, we expected this screen to contain substantial noise. Also, we expected positive signals (i.e. GI candidates) arising from a pan-cancer screen would represent strong signals, and GI candidates appearing in both pan-cancer and cancer type-specific screens to be high-confidence candidates. Therefore, we rationalised the inclusion of the pan-cancer dataset, which resulted in the analysis of ten datasets.

For the pan-cancer dataset and each of the nine cancer type-specific datasets, we used GRETTA [6, 30] to identify *KMT2D*^LOF^ and *KMT2D*^WT^ DepMap cancer cell lines (Additional file 5: Fig. S1A; Additional file 6: Table S5A; Methods). Next, we compared the *KMT2D* mRNA and protein expression between the *KMT2D*^WT^ and *KMT2D*^LOF^ cell lines within each dataset. *KMT2D* mRNA expression was significantly higher in *KMT2D*^LOF^ lines than in *KMT2D*^WT^ lines in the pan-cancer and UCEC datasets (Welch’s *t*-test *p*-value < 0.05; Additional file 5: Fig. S1B). On the other hand, KMT2D protein expression in *KMT2D*^LOF^ cell lines was significantly lower in the pan-cancer dataset and trended lower than in *KMT2D*^WT^ cell lines across all nine cancer type-specific datasets for which data were available, including COAD/READ, LUSC, SCLC, STAD, and UCEC (Fig. 2B). The discordance in protein and mRNA expression is well documented for many genes [101–103] but has not yet been documented for *KMT2D*. We did not attempt to re-construct the isoforms being expressed or otherwise assess the extent to which the mRNA expressed might encode function, judging that this to be beyond the scope of this study. Our data showed that *KMT2D*^LOF^ alterations were associated with lower KMT2D protein expression so, from this, we infer that KMT2D activities are reduced or lost in most cancer types, as expected. We also compared the relative global levels of active histone marks captured by DepMap, namely H3K4me1, H3K4me2, and H3K27ac, which are known to be reduced in KMT2D-deficient cells [86, 104–107]. Briefly, DepMap utilises an MS-based method to profile relative changes in levels of histone modifications, such as methylation and acetylation, and allows quantification of marks as combinations (e.g. H3K27ac1K36me0), which is not generally possible with antibody-based methods [32]. We found a significant reduction of global H3K4me1 and H3K4me2 levels in *KMT2D*^LOF^ cell lines of the pan-cancer and COAD/READ-specific datasets compared to their respective *KMT2D*^WT^ cell lines (Welch’s *t*-test *p*-value < 0.05; Additional file 5: Fig. S1C). B-NHL *KMT2D*^LOF^ cell lines also had significantly reduced levels of H3K4me2 compared to *KMT2D*^WT^ cell lines (Welch’s *t*-test *p*-value < 0.05; Additional file 5: Fig. S1C), and both H3K4me1 and H3K4me2 levels trended lower in *KMT2D*^LOF^ BLCA, SCLC, and UCEC cell lines, consistent with a reduction in KMT2D activity. Interestingly, the combined histone mark levels H3K27ac1K36me0 were significantly higher in B-NHL *KMT2D*^LOF^ cell lines compared to *KMT2D*^WT^ cell lines (Welch’s *t*-test *p*-value < 0.05; Additional file 5: Fig. S1C), which suggests that KMT2C, EP300, or CREBBP activity may be compensating to maintain homeostasis of transcriptional activity disrupted by KMT2D loss [79]. Altogether, these results are consistent with the notion that KMT2D function is reduced in our selected *KMT2D*^LOF^ cell lines and that such cell lines are suitable for our in silico genetic screening analyses.

Finally, using GRETTA [30], we performed differential lethality analyses to predict *KMT2D* GIs. In this analysis, a gene KO that led to significantly higher lethality probability in the *KMT2D*^LOF^ cell lines compared to *KMT2D*^WT^ cell lines indicated a potential SL interactor, whereas a KO that led to significantly lower lethality probability in *KMT2D*^LOF^ cell lines indicated a potential AL interactor. Using this method, we predicted 4396 GIs with 3692 unique GIs across all screens (Additional file 6: Table S5B; Methods). In context-specific screens, most predicted *KMT2D* GIs (~ 80%) were unique to the cancer type in which they were screened (Fig. 2C), which is consistent with the view that GIs are highly context-dependent [108]. As expected, 46% of GIs predicted in the pan-cancer screen were also found in context-sensitive screens, given that cell lines in the pan-cancer screens are made up of several cancer types. The largest overlap was seen between the pan-cancer and COAD/READ screens, which is likely due to the large proportion of *KMT2D*^LOF^ cell lines that are COAD/READ (5/16 cell lines).

To highlight pathways that are associated with candidate *KMT2D* GIs, we annotated the SL and AL candidates with the biological processes in which they are involved. The SL candidates were associated with 67 biological processes (Methods; Additional file 6: Table S5C). Of these, three (4%) were associated with mitotic processes and homologous recombination, five (7%) were associated with metabolic processes, and ten (15%) were associated with T-cell cytotoxicity and immune cell response (Additional file 6: Table S5C). The AL candidates were associated with 301 biological processes. Five (~ 2%) were associated with mitotic processes, 26 (9%) with metabolism, and four (1%) with immune response (Additional file 6: Table S5C). To determine whether these biological processes were more abundantly represented in SL or AL interactors, we performed an equality of proportion analysis [109] to determine whether the proportion of mitotic, metabolic, and immune-related terms was more abundant among SL or AL interactors. We found the proportion of immune-related terms was significantly higher in SL interactors than in AL interactors (equality of proportions *p*-value < 0.0001); however, no difference was found for mitotic or metabolic-associated terms. These results indicate that genes associated with immune response were more abundant among SL candidates. Therefore, *KMT2D*^LOF^ cells may be vulnerable to perturbations of immune response-associated pathways.

### GI prediction scores and target tractability assignments prioritise therapeutically promising *KMT2D* GIs

Following the method introduced by Behan et al. [8] to determine high-quality *KMT2D* interactors and prioritise those that might be candidate drug targets, we combined two classification systems, one based on GI prediction statistics and the other based on drug tractability (i.e. the likelihood of identifying a drug targeting the protein encoded by the genetic interactor; summarised in Table 2; Methods). We classified GI candidates into three tiers based on statistical significance (GI tiers I, II, and III), where GI tier I candidates represented the most statistically significant group. The second classification was based on drug tractability (drug groups I, II, and III), with group I containing candidates that are targets of existing drugs. Finally, we combined the GI tiers and drug tractability groups to create four priority classes (A, B, C, and D), with priority class A representing the highest level of GI prediction significance and the highest evidence for drug tractability.

**Table 2:** Definitions for prioritising GI candidates. (Top) GI tiers are based on in silico screen statistics. (Middle) Drug tractability groups are based on OpenTarget definitions. (Bottom) Priority classes combine GI tier and drug group classifications, with class A having the highest priority and D having lowest priority candidates

**GI prediction tier**	**Adjusted Mann Whitney** ***U*-test** ***p*-value**	**Log2 fold change of absolute median lethality probabilities**	**Minimum WT/LOF group median lethality probability**	
I	< 0.01	> 2	> 0.5	
II	< 0.05	> 2	> 0.5	
III	< 0.05	> 2	None	
**Drug tractability group**	**Small molecule tractability**	**Antibody tractability**	**PROTAC tractability**	**Other clinical tractability**
I	Clinical precedence	Clinical precedence	Clinical precedence	Clinical precedence
II	Discovery precedence	Predicted tractable (high confidence)	Literature precedence	None
III	Predicted tractable	Predicted tractable (medium to low confidence)	Discovery opportunity	None
**Priority class**	**GI prediction tier**	**Drug tractability group**		
A	I	I		
B	I	II		
C	I	III/None		
D	II/III	I/II/III/None		

We identified three priority class A candidates—namely the SL interactors *MDM2*, *TUBA1B* (COAD/READ), and *NDUFB5* (STAD) and the AL interactor *CDK6* (in the pan-cancer dataset)—that encode targets of approved anticancer drugs or that are the focus of drug development efforts (Fig. 2D–F; Additional file 6: Table S5D) [36]. *MDM2* encodes a negative regulator of p53 that is amplified and overexpressed in 9% of colorectal cancers [110]. *TUBA1B* encodes α-tubulin, a microtubule component that functions in cell motility, adhesion, and cell division. In hepatocellular carcinoma, elevated TUBA1B expression has been associated with a superior response to ICI therapy [111]. In COAD cases, elevated expression was associated with improved overall survival and with CD8 + pre-exhausted T-cells [112]. Interestingly, both MDM2 and TUBA1B have been associated with the DNA damage response [113, 114], a function also associated with KMT2D [72]. *NDUFB5* encodes a subunit of complex I, which plays a role in oxidative phosphorylation [115, 116], a pathway known to be dysregulated in KMT2D-deficient lung adenocarcinomas and Kabuki syndrome patients [117, 118].

Priority class B candidates (targets without drugs in clinical development but with high-confidence evidence supporting target tractability, such as having high-quality ligand structures for pharmaceutical development [36]) include seven genes, specifically the SL interactors *WRN* (pan-cancer and COAD/READ), *MRPS17* (ESCA), and *MTG1* (STAD) and the AL interactors *ZNF217* (COAD/READ), *RAB7A* (ESCA), and *ACTR5* (HNSC; priority class B; Fig. 2D–F). *WRN* encodes a DNA helicase and nuclease that functions in DNA replication, repair, and transcription [119–121] and has recently been identified as a SL vulnerability of MMR-deficient MSI cancers, including some colorectal, endometrial, gastric, and ovarian cancers [8, 60–63, 122]. Although *WRN* was categorised as a drug target group II candidate by the Open Target Platform [36] (Methods), there are currently several drug development programs underway to target WRN [123, 124] in MSI cancer; therefore, we re-classified it as a drug target group I and priority class A gene.

Priority class C candidates consisted of interactors without drugs in clinical development and medium to low-confidence evidence supporting tractability. They included 19 genes, including the SL interactors *FBXW11* (LUSC) and *TFB1M* (STAD; priority class C; Fig. 2D–F). Interestingly, FBXW11 is part of an E3 ubiquitin ligase complex involved in ubiquitination and proteasomal degradation in tumourigenesis signalling pathways [125] and has been shown to co-purify with the polycomb repressive complex 2 (PRC2) members EED [126], SUZ12, and EZH2 [125], which play an antagonistic role to COMPASS complex members by depositing the repressive histone modification mark H3K27me3 [84]. TFB1M is a mitochondrial transcription specificity factor that maintains homeostasis of oxidative phosphorylation and glycolysis [127, 128], pathways that have been shown to be dysregulated in KMT2D-deficient lung cancers [117], epithelial cells, and Kabuki Syndrome patients [118]. Notably, KMT2D-deficient lung cancer cells depend on proper glycolytic activities for survival [117], which is compatible with the notion that loss of *TFB1M* may be lethal for KMT2D-deficient cells.

### Glycolytic genes are dysregulated in TCGA-STAD *KMT2D*^LOF^ cases

**Fig. 3:**
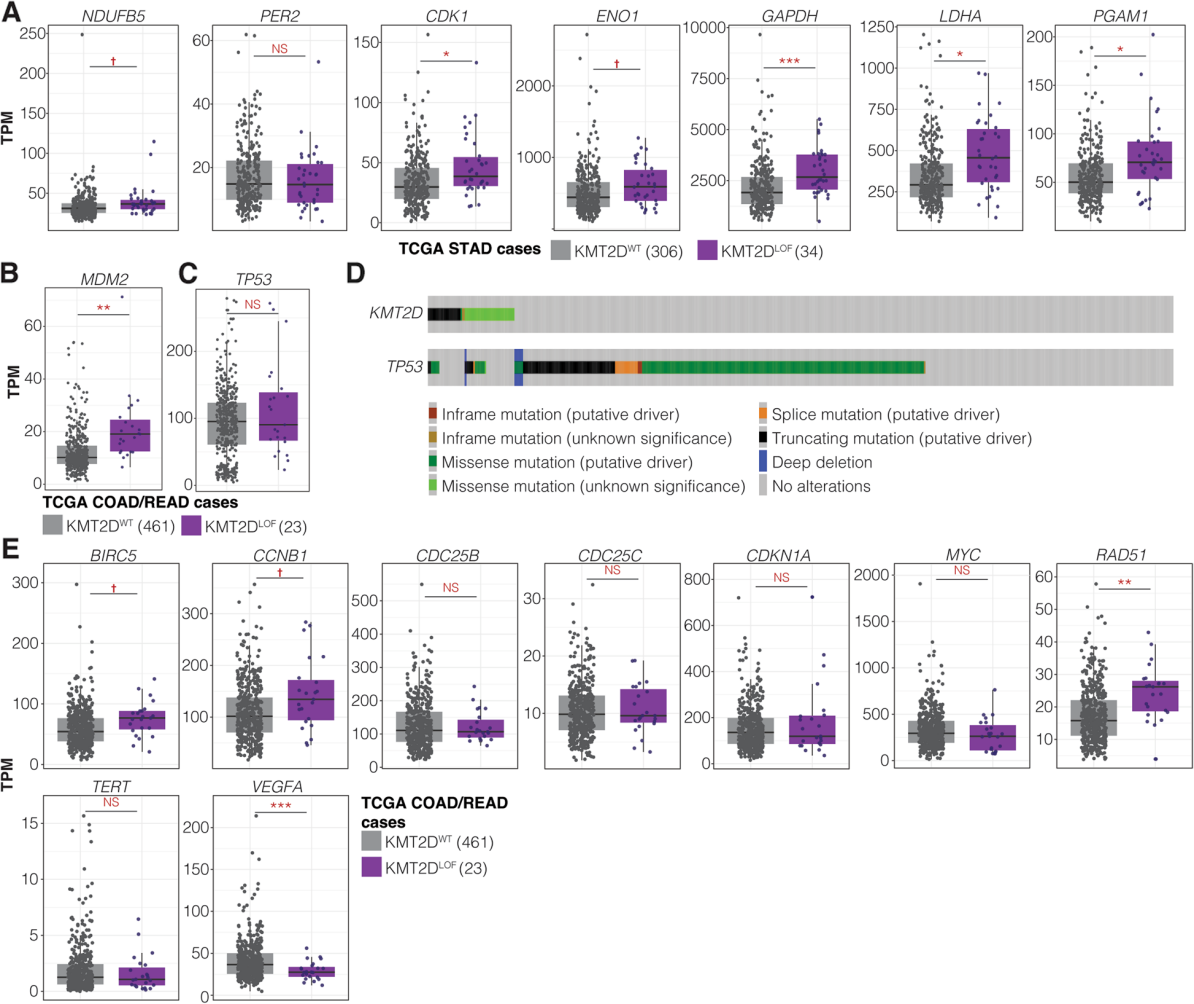
*KMT2D*^LOF^ TCGA cases may be vulnerable to *NDUFB5* and *MDM2* perturbations. **A** *NDUFB5* and glycolytic genes in TCGA-STAD cases. **B,C** *MDM2* (**B**) and *TP53* (**C**) mRNA expression in TCGA-COAD/READ *KMT2D*^LOF^ cases and *KMT2D*.^WT^ cases. **D** cBioPortal oncoprint of *KMT2D* and *TP53* mutations found in 565 TCGA-COAD/READ cases [39, 40]. Fisher’s exact test *p*-value < 0.01 and log_2_OR = − 1.25. **E** mRNA expression of genes regulated by p53 in TCGA-COAD/READ cases. BH-corrected Welch’s *t*-test *p*-value † < 0.1, ** < 0.01, *** < 0.001, and NS > 0.05

Given that SL candidates *NDUFB5* in the STAD screen; *MDM2* and *TUBA1B* in the COAD/READ screen; and *WRN* in the COAD/READ and pan-cancer screens were identified as the most promising therapeutic targets (priority class A) for *KMT2D*^LOF^ cancers, we sought to understand the relationship between *KMT2D* and these genes further. *NDUFB5* encodes a subunit of the mitochondrial respiratory chain enzyme Complex I (NADH:ubiquinone oxidoreductase), which functions to create a proton gradient for ATP synthesis through oxidative phosphorylation (OXPHOS) [115, 116]. Interestingly, studies by Alam et al. [117] in a lung cancer model and Maitituoheti et al. [129] in a melanoma model showed that KMT2D deficiency led to dysregulation of OXPHOS and glycolytic activity, conferring a vulnerability to perturbation in glycolytic functions. Alam et al. [117] also demonstrated that KMT2D loss impaired epigenetic signals, which reduced PER2 expression, a transcriptional repressor of multiple glycolytic genes. Therefore, we investigated whether *KMT2D*^LOF^ alterations were associated with differences in the expression of *NDUFB5*, *PER2*, and PER2-regulated glycolytic genes in TCGA-STAD cases. We found a trend towards increased *NDUFB5* mRNA expression (BH-corrected Welch’s *t*-test *p*-value < 0.1; Fig. 3A; Additional file 7: Table S6; Methods) in *KMT2D*^LOF^ cases compared to *KMT2D*^WT^ cases. Although we did not find a difference in *PER2* expression, PER2-regulated glycolytic genes, namely *CDK1*, *ENO1*, *GAPDH*, *LDHA*, and *PGAM1* [117], all showed either a trend towards or a statistically significant mRNA expression elevation in *KMT2D*^LOF^ cases compared to *KMT2D*^WT^ cases, consistent with results demonstrated by Alam et al. [117] in a *Kmt2d* knockdown mouse lung cancer cell line (Fig. 3A; Additional file 7: Table S6; Methods). Furthermore, we found that, in addition to *NDUFB5*, all GI tier I candidates in the STAD screen, namely *ATP2B1* [130], *DARS2* [131], *MTG1* [132], *NUBPL* [133], and *TFB1M* [127, 128, 134], were SL interactors and were associated with mitochondrial and metabolic functions (Additional file 6: Table S5). Our results further support the notion that *KMT2D* may play a role in metabolism and that *KMT2D*^LOF^ alterations in STAD cases may also confer a vulnerability to OXPHOS or glycolytic perturbations.

### MDM2 is overexpressed in TCGA-COAD/READ *KMT2D*^LOF^ cases

*MDM2* encodes an E3 ligase that labels the tumour suppressor p53 for degradation [135]. MDM2 has been shown to be aberrantly upregulated across several cancers, including COAD/READ, leading to enhanced degradation of p53, reduced p53 activities, and disease progression [135, 136]. Although MDM2 has not been linked to KM2TD, MDM2 overexpression has been shown to stabilise and enhance KMT2A accumulation on the chromatin and promote H3K4me3 deposition [137], and KMT2D has been shown to interact with p53 [91, 92]. Given that we predicted a SL interaction between *KMT2D* and *MDM2* in the COAD/READ GI screen and observed protein–protein interaction between KMT2D and p53, we therefore sought to better understand the relationship between *KMT2D*, *MDM2*, and *TP53*. We first queried TCGA-COAD/READ data to compare the transcriptome profiles of untreated *KMT2D*^LOF^ cases to *KMT2D*^WT^ cases. We found that COAD/READ *KMT2D*^LOF^ cases had significantly higher *MDM2* mRNA expression compared to *KMT2D*^WT^ cases (Welch’s *t*-test *p*-value < 0.01; Fig. 3B; Additional file 8: Table S7); however, there was no difference in *TP53* expression (Fig. 3C). Interestingly, mutations affecting *KMT2D* and *TP53* were significantly mutually exclusive (Fisher’s exact test *p*-value < 0.01 and log_2_ odds ratio [OR] − 1.25; Fig. 3D; Additional file 8: Table S7), indicating that p53 functions are likely intact in most *KMT2D*^LOF^ cases. Mutual exclusivity between *KMT2D* and *MDM2* was not analysed due to the low number of cases with *MDM2* mutations (*N* = 7). To determine whether the significantly higher expression of *MDM2* in *KMT2D*^LOF^ cases resulted in reduced p53 function, we compared the expression of genes whose expression is repressed by p53, namely *BIRC5*, *CCNB1*, *CDC25B*, *CDC25C*, *CDKN1A*, *MYC*, *RAD51*, *TERT*, and *VEGFA* [138, 139]. *RAD51* expression was significantly elevated (BH-corrected Welch’s *t*-test *p*-value < 0.01), and *BIRC5* and *CCNB1* expression showed a trend towards increase in *KMT2D*^LOF^ cases compared to *KMT2D*^WT^ cases (BH-corrected Welch’s *t*-test *p*-value < 0.1; Fig. 3E). From this, we inferred that p53 activities may be reduced in *KMT2D*^LOF^ cases. No difference in expression of *CDC25B*, *CDC25C*, *CDKN1A*, and *MYC* was observed. Conversely, *VEGFA* expression was significantly reduced in *KMT2D*^LOF^ cases compared to *KMT2D*^WT^ cases (BH-corrected Welch’s *t*-test *p*-value < 0.1; Fig. 3E), which is consistent with previous observations that showed p53 can both repress and promote *VEGFA* expression [140, 141]*.* Altogether, our results show that TCGA-COAD/READ *KMT2D*^LOF^ cases have increased *MDM2* expression, which may result in reduced or dysregulated p53 activities, and thus highlight MDM2 as a potentially attractive candidate for therapeutic targeting in COAD/READ *KMT2D*^LOF^ cases.

### *KMT2D*^LOF^ MSI TCGA-COAD/READ cases have significantly elevated ICI response indicators

**Fig. 4:**
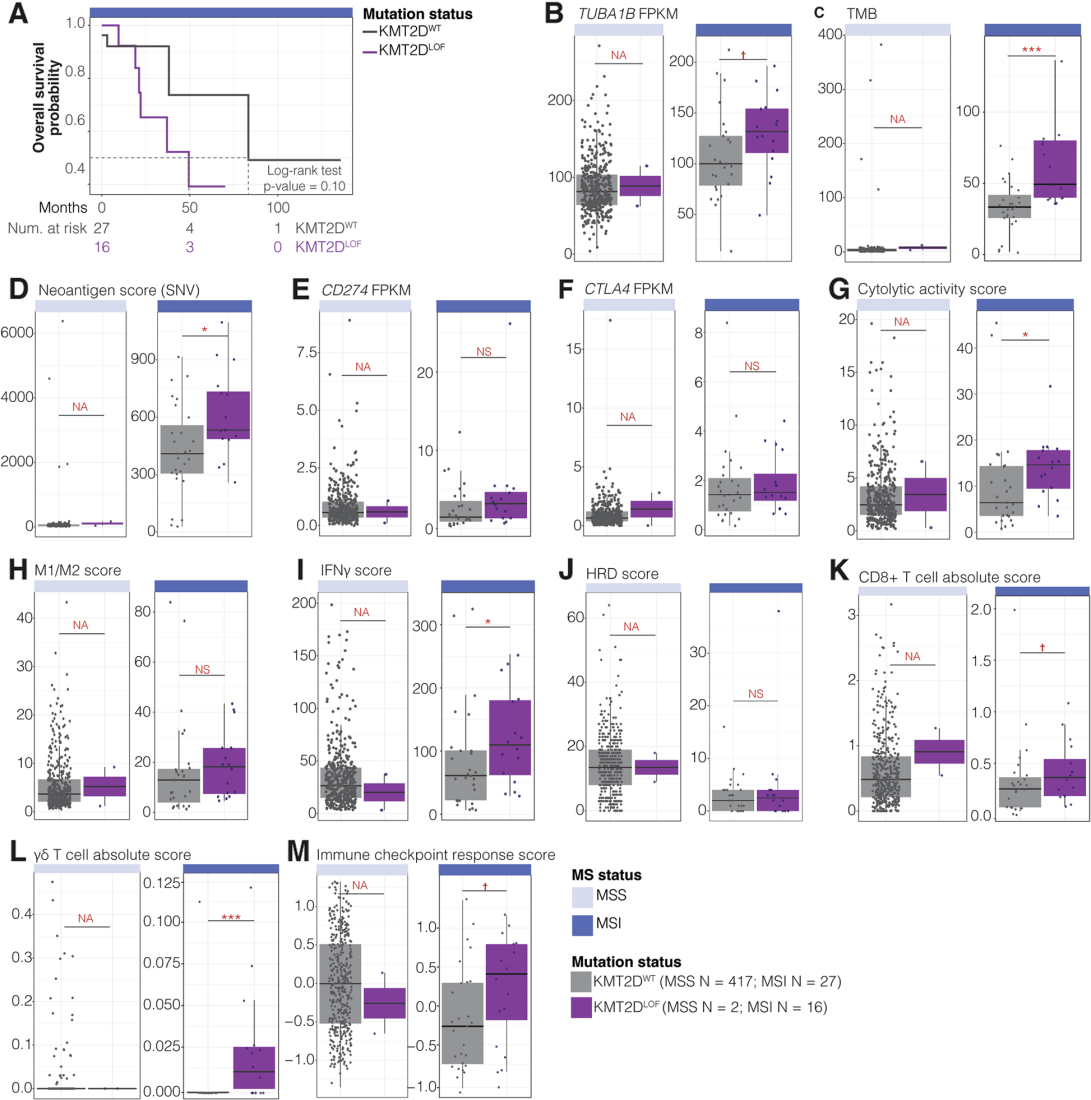
*KMT2D*^LOF^ TCGA-COAD/READ cases may be vulnerable to ICI treatment. **A **Kaplan–Meier curves (top) and risk table (bottom) of overall survival of MSI TCGA-COAD/READ *KMT2D*^WT^and *KMT2D*^LOF^ cases. **B-M** A comparison of ICI response markers between TCGA-COAD/READ *KMT2D*^WT^ and *KMT2D*^LOF^ MSI/MSS cases. ICI response markers included: (B) *TUBA1B* mRNA expression, (C) TMB, (D) neoantigen score, (E-F) mRNA expression of *CD274* and *CTLA4*, (G) cytolytic activity score, (H) M1/M2 macrophage score, (I) IFN𝛾 score, (J) HRD score, (K-L) CD8 + and γδ T-cell infiltration score, and (M) PredictIO ICI response score. Welch’s t-test *p*-value † < 0.1, * < 0.05, ** < 0.01, *** < 0.001, and NS > 0.1. MSS cases were not analysed statistically (NA) due to the small number of *KMT2D*^LOF^ cases

MSI is an approved tissue/site-agnostic biomarker for ICI treatments [142, 143]. A recently completed analysis of KEYNOTE-177 (in April of 2022), a randomised, open-label, phase-three study comparing MSI/MMR-deficient metastatic colorectal cancer cases treated with pembrolizumab versus chemotherapy, showed that although pembrolizumab-treated patients had fewer treatment-related adverse effects and a progression-free survival improvement was noted, no significant difference in overall survival between the two treatment groups was observed [144]. Therefore, there is a need to improve ICI treatment stratification. Given that we identified several SL candidates with roles in T-cell cytotoxicity and the immune response (Additional file 6: Table S5C), identified *TUBA1B* as a priority class A target in the COAD/READ screen, and showed that MSI can elicit specific vulnerabilities in *KMT2D*^LOF^ cancer cell lines; we sought to understand the impact of *KMT2D*^LOF^ on immune markers in MSS and MSI COAD/READ cases and whether *KMT2D* mutational status might be relevant in the context of ICI treatment. To this end, we profiled the clinical, mutational, and transcriptomic profiles of untreated COAD/READ MSI cases from TCGA. We identified 43 MSI COAD/READ cases (27 *KMT2D*^WT^ MSI and 16 *KMT2D*^LOF^ MSI cases; Methods) and observed a trend towards lower overall survival probability in *KMT2D*^LOF^ MSI cases compared to *KMT2D*^WT^ MSI cases (log-rank test *p*-value = 0.10; Fig. 4A; Additional file 9: Table S8; Methods). A survival analysis was not performed for MSS cases due to the small sample size of *KMT2D*^LOF^ cases (417 *KMT2D*^WT^ MSS and two *KMT2D*^LOF^ MSS cases). This supports previous observations showing that KMT2D deficiency leads to inferior survival outcomes compared to KMT2D-proficient patients with lymphoma, lung, and breast cancers [145–148].

Next, in the TCGA-COAD/READ cohort, we characterised and compared the expression of *TUBA1B*, as an elevated expression has been associated with superior response to ICI therapy [111], and markers of immune response, including TMB [149, 150], neoantigen scores [45], *CD274/PD-L1* and *CTLA4* expression [151], cytolytic activity scores [54, 152], M1/M2 macrophage scores [55, 153], interferon-gamma (IFNγ) signalling scores [154], and homologous recombination deficiency (HRD) scores [155]. Due to a small *KMT2D*^LOF^ MSS sample size, statistical analyses of MSS cases were not performed for any of the immune markers. However, in MSI cases, we found a trend towards higher *TUBA1B* expression in *KMT2D*^LOF^ MSI cases compared to *KMT2D*^WT^ MSI cases (Welch’s *t*-test *p*-value = 0.057; Fig. 4B; Additional file 9: Table S8; Methods). We also observed a significantly higher TMB in *KMT2D*^LOF^ MSI compared to *KMT2D*^WT^ MSI cases (Welch’s *t*-test *p*-value < 0.001; Fig. 4C; Additional file 9: Table S8; Methods), which supports previous observations showing that KMT2D deficiency led to increased genomic instability in mouse embryonic fibroblast and human colorectal cell line models [72]. A comparison of mutational signatures using the Catalogue Of Somatic Mutations In Cancer (COSMIC) indicated that the single base pair substitution (SBS) signatures associated with MMR deficiency were not significantly different between *KMT2D*^LOF^ and *KMT2D*^WT^ MSI cases (BH-corrected Welch’s *p*-value > 0.05; Additional file 10: Table S9; Methods). However, several other mutational signatures, namely SBS1, SBS5, SBS22, and SBS54, were elevated in *KMT2D*^LOF^ MSI cases compared to *KMT2D*^WT^ MSI cases (uncorrected Welch’s *p*-value < 0.05 but > 0.05 when using BH correction; Additional file 11: Fig. S2A). SBS54 is a possible sequencing artefact, whereas SBS22 is associated with aristolochic acid exposure [43, 156]. Interestingly, increased SBS1 and SBS5 mutation rates are associated with increased mutational burden and have been linked to clock-like signatures [43, 156]. However, a comparison of chronological age did not show a significant difference between *KMT2D*^LOF^ and *KMT2D*^WT^ cases (Welch’s *t*-test *p*-value > 0.05; Additional file 11: Fig. S2B). SBS1 arises from an endogenous mutational process that generates guanine:thymine mismatches in double-stranded DNA due to the deamination of 5-methylcytosine to thymine [43, 156, 157]. Therefore, the elevated SBS1 signature in *KMT2D*^LOF^ cases indicates that LOF alterations in *KMT2D* may induce endogenous mismatches in addition to those acquired due to MSI. We also observed significantly higher neoantigen scores in *KMT2D*^LOF^ MSI cases than in *KMT2D*^WT^ MSI cases in COAD/READ (Welch’s *t*-test *p*-value < 0.05; Fig. 4D; Additional file 9: Table S8; Methods). Next, we compared the expression of *CD274* (encoding PD-L1) and *CTLA4*. Although not statistically significant, we observed a trend towards higher *CD274* expression in *KMT2D*^LOF^ MSI cases (4.38 mean FPKM) compared to *KMT2D*^WT^ MSI cases (2.67 mean FPKM; Welch’s *t*-test *p*-value = 0.15; Fig. 4E). No such trend was observed in *CTLA4* expression (Welch’s *t*-test *p*-value = 0.45; Fig. 4F; Additional file 9: Table S8; Methods). Furthermore, we also compared the difference in immune cytolytic activity scores, a measure for anti-tumour immune cell activity [54, 152], and found that *KMT2D*^LOF^ MSI cases had significantly higher immune cytolytic activity scores compared to *KMT2D*^WT^ MSI cases (Welch’s *t*-test *p*-value < 0.05; Fig. 4G; Additional file 9: Table S8; Methods). Together with the trending increase in *CD274* expression, our results may indicate that *KMT2D*^LOF^ MSI COAD/READ cancers have increased T-cell infiltration and anti-tumour immune cell activity compared to *KMT2D*^WT^ MSI cases. We next compared M1/M2 macrophage scores. While we did not find a statistically significant difference, M1/M2 scores did show elevated trends in *KMT2D*^LOF^ MSI COAD/READ cases compared to *KMT2D*^WT^ MSI COAD/READ cases (Fig. 4H; Additional file 9: Table S8; Methods). Next, we calculated the IFNγ signalling scores [56] and found that *KMT2D*^LOF^ MSI cases had significantly higher scores than *KMT2D*^WT^ MSI (Welch’s *t*-test *p*-value < 0.05; F 4g. 4I; Additional file 9: Table S8; Methods). We also compared HRD scores and did not observe a significant difference in HRD scores between *KMT2D*^LOF^ MSI and *KMT2D*^WT^ MSI cases (Fig. 4J; Additional file 9: Table S8; Methods).

Given that tumour immune microenvironment composition has also been shown to be predictive of ICI response [158], we used CIBERSORTx [57] to estimate the extent of immune cell infiltration in TCGA COAD/READ cases and compared the difference in predicted immune cell composition between *KMT2D*^LOF^ MSI and *KMT2D*^WT^ MSI cases. We found a higher predicted abundance of cytotoxic CD8^+^ T-cells and γδT-cells, a cytotoxic effector that produces pro-inflammatory cytokines [159, 160], in *KMT2D*^LOF^ MSI compared to *KMT2D*^WT^ MSI COAD/READ cases (Welch’s *t*-test *p*-value < 0.1 and *p* < 0.001, respectively; Fig. 4K,L; Additional file 9: Table S8; Methods). However, we did not observe differences in the proportion of other immune cell populations (Additional file 11: Fig. S2C). These results support the notion that MSI COAD/READ cases harbouring *KMT2D*^LOF^ alterations may have more T-cell infiltration than *KMT2D*^WT^ cases.

Finally, to predict whether *KMT2D*^LOF^ MSI COAD/READ cases might respond more favourably to ICI treatment than *KMT2D*^WT^ MSI COAD/READ cases, we calculated their ICI response scores using PredictIO [58]. Briefly, PredictIO uses a signature based on 100 genes that best predict response to ICIs across various cancer types [58]. While we did not find a statistically significant difference, we did observe a higher trend in ICI response scores in *KMT2D*^LOF^ MSI cases (Welch’s *t*-test *p*-value < 0.1; Fig. 4M; Additional file 9: Table S8; Methods), indicating that *KMT2D*^LOF^ MSI COAD/READ cases may exhibit a more favourable response when treated with ICIs than *KMT2D*^WT^ MSI cases. Overall, despite the small number of MSI cases challenging our statistical analyses, we were still able to show, for the first time, that *KMT2D*^LOF^ alterations in MSI cases appear to be associated with significantly higher TMB than MSI alone (Welch’s *t*-test *p*-value < 0.001; Fig. 4C). Furthermore, we showed that *KMT2D*^LOF^ MSI cases had significantly higher expression of neoantigens, immune-activation signatures, cytotoxic T-cell proportions, and ICI-response scores, all consistent with the possibility of more favourable ICI responses compared to *KMT2D*^WT^ MSI COAD/READ cases.

### *KMT2D*^LOF^ MSI alterations may be associated with elevated trends in immune response indicators in the POG and MSK-IMPACT cohorts

**Fig. 5:**
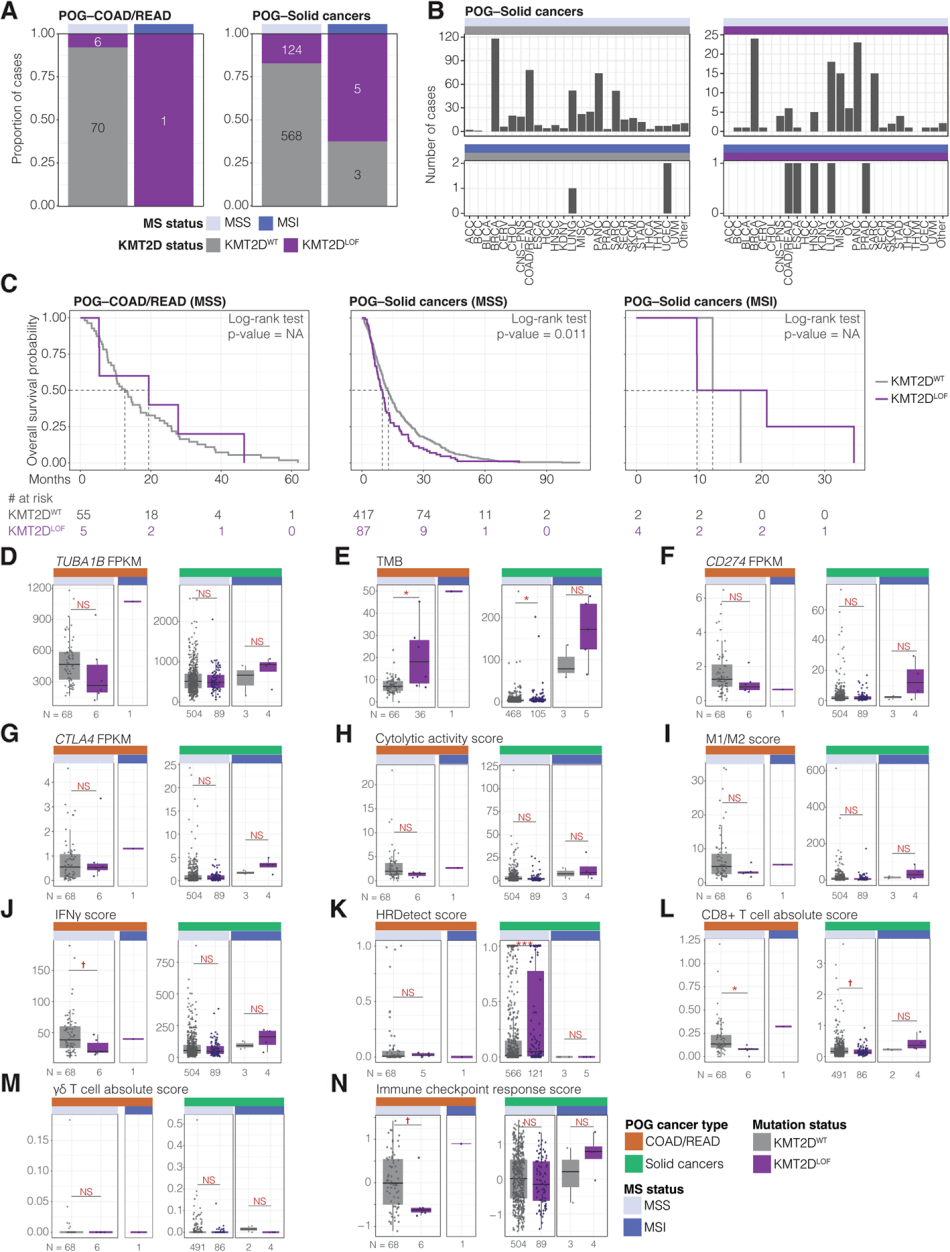
ICI response markers are elevated in *KMT2D*^LOF^ POG-MSI solid cancer cohort cases. **A** Proportion of advanced and metastatic POG-COAD/READ (left) and all solid cancer (right) cases with MSI and *KMT2D*^LOF^alterations.** B** Number of cases by cancer type found in the POG-solid cancer cohort in panel A. **C** Kaplan–Meier curves (top) and risk table (bottom) comparing overall survival of *KMT2D*^LOF^ cases to *KMT2D*^WT^ cases. **D-N** A comparison of ICI response markers between *KMT2D*^WT^ and *KMT2D*^LOF^ MSI/MSS cases in POG-COAD/READ and solid cancer cohorts. ICI response markers included: (D) *TUBA1B* mRNA expression, (E) TMB, (F-G) RNA expression of *CD274* and *CTLA4*, (H) cytolytic activity score, (I) M1/M2 macrophage score, (J) IFN𝛾 score, (K) HRDetect score (L-M) CD8 + and γδ T-cell infiltration score, and (N) PredictIO ICI response score. Welch’s t-test *p*-value † < 0.1, * < 0.05, ** < 0.01, *** < 0.001, and NS > 0.1. No statistical analysis was performed for the MSI POG-COAD/READ case, due to small sample size (*N* < 3)

We next analysed two cohorts, the advanced and metastatic cancer patients from the Personalized OncoGenomics (POG) Project at BC Cancer (NCT02155621 [14, 15]) and advanced cancer patients treated with ICI from MSK-IMPACT (NCT01775072 [13]). POG and MSK-IMPACT cases differ from untreated TCGA cases, as they are at an advanced stage, are incurable, and have been heavily treated. They closely represent the patient populations typically receiving second- or third-line immunotherapy. We first identified COAD/READ cases harbouring *KMT2D*^LOF^ mutations and their MS status. However, the number of MSS and MSI cases harbouring *KMT2D*^LOF^ alterations was small (six and one case, respectively; Fig. 5A). Therefore, we also included all solid cancers, which included COAD/READ and 26 other cancer types (Fig. 5A,B), and identified 124 *KMT2D*^LOF^ MSS cases and five *KMT2D*^LOF^ MSI cases (Fig. 5A). Similar to the trends seen in TCGA-COAD/READ MSI cohort, we computed statistically significant lower overall survival probability in POG *KMT2D*^LOF^ MSS solid cancer cases than in *KMT2D*^WT^ MSS solid cancer cases (Log-rank test *p*-value = 0.01; Fig. 5C; Additional file 12: Table S10). However, we did not conduct survival analyses in COAD/READ MSS and MSI solid cancer cases due to limited case numbers. MSK-IMPACT data included few *KMT2D*^LOF^ MSS and MSI cases, and no significant difference in survival was observed (Additional file 13: Fig. S3A-C). Our analysis thus showed that, at least in the MSS heavily treated POG patients, *KMT2D*^LOF^ cases appeared to have reduced overall survival probabilities compared to *KMT2D*^WT^ cases, although this did not reach significance, as was also seen in untreated TCGA cases.

Next, we analysed ICI response markers in POG and MSK-IMPACT cases (Fig. 5D–N; Additional file 13: Fig. S3D and Additional file 14: Fig. S4A-C; Additional file 12: Table S10; Methods), as we did with TCGA cases. For the POG cohort, no comparisons were made in COAD/READ MSI cases for any ICI response markers since there was only one case harbouring *KMT2D*^LOF^ alterations, as mentioned above. In the MSK-IMPACT cohort, only TMB was estimated since this study sequenced a targeted panel of genes and lacked transcriptome data [13]. In POG and in MSK-IMPACT data for COAD/READ and solid cancers, we saw significantly higher TMB in *KMT2D*^LOF^ MSS cases compared to *KMT2D*^WT^ MSS cases (Welch’s *t*-test *p*-value < 0.05; Fig. 5E; Additional file 13: Fig. S3D). Furthermore, in the MSK-IMPACT cohort, compared to *KMT2D*^WT^ MSI solid cancer cases, TMB was also significantly elevated in *KMT2D*^LOF^ MSI solid cancer cases (Welch’s *t*-test *p*-value < 0.05; Additional file 13: Fig. S3D). In the POG solid cancer MSI cases and MSK-IMPACT COAD/READ cases, *KMT2D*^LOF^ cases trended towards elevated TMB compared to *KMT2D*^WT^ cases (Fig. 5E; Additional file 13: Fig. S3D). We also saw trends in the same direction for *TUBA1B*, *CD274*, and *CTLA4* expression (Fig. 5D, 5, 5); M1/M2 scores (F 5g. 5I); IFNγ scores (Fig. 5J); CD8 + T-cell scores (Fig. 5L); and ICI response scores (Fig. 5N) in *KMT2D*^LOF^ MSI solid cancer compared to *KMT2D*^WT^ MSI solid cancer cases. The substantially smaller sample sizes (a total of one *KMT2D*^LOF^ case in the COAD/READ MSI cases and five in the solid cancer MSI cases) resulted in insufficient power to detect significant differences in the POG cohort. Even so, our findings are consistent with a similar effect of *KMT2D*^LOF^ in the POG and MSK-IMPACT cohorts as we observed in TCGA. Our results reinforce the notion that MSI cases with *KMT2D*^LOF^ alterations may respond more favourably to ICI treatment than *KMT2D*^WT^ MSI cases and that consideration of *KMT2D* mutational status may improve patient stratification for ICI treatment.

### *KMT2D* mutational status significantly stratifies MSI COAD/READ cell lines sensitive to WRN inhibitors

**Fig. 6:**
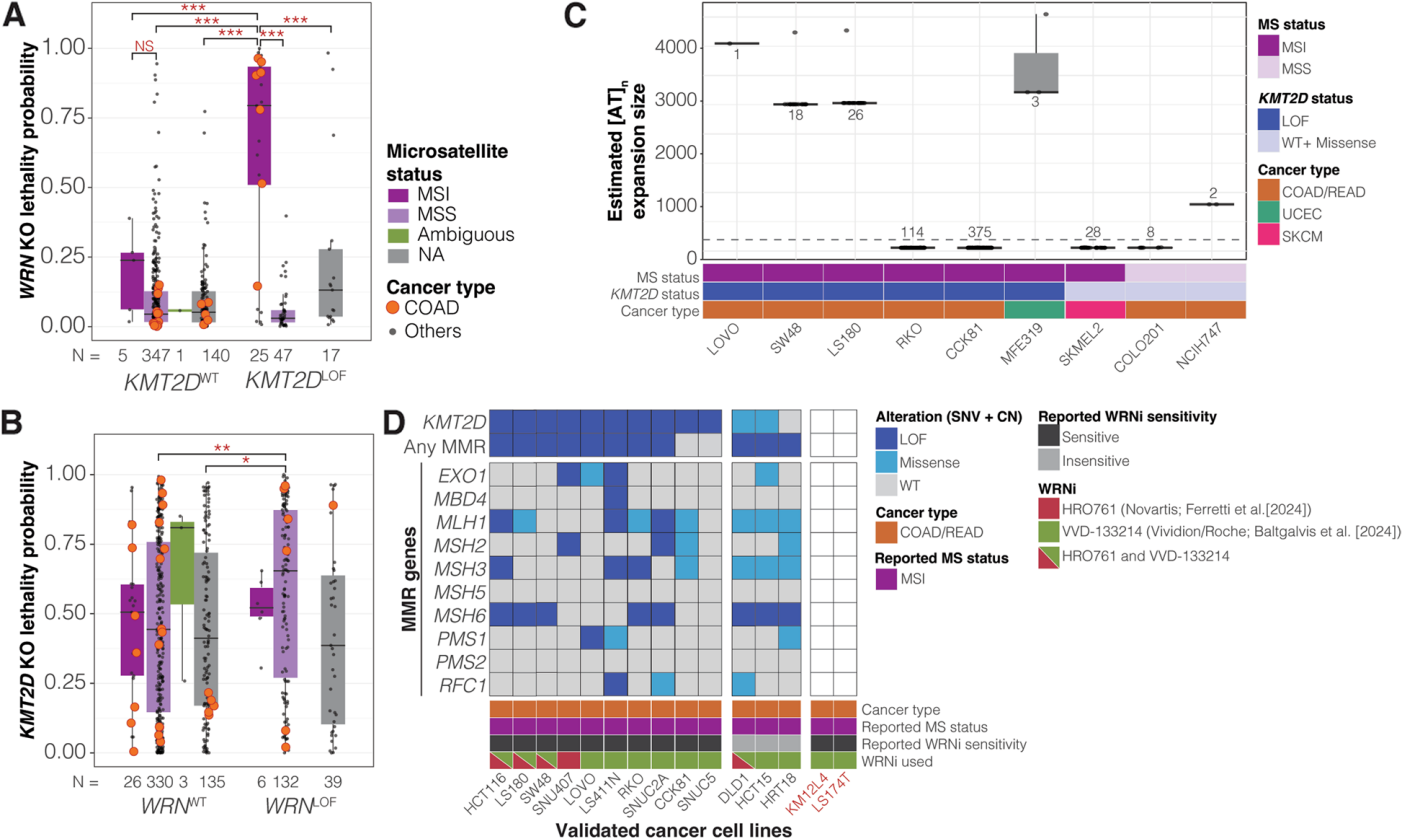
*KMT2D* mutational status stratifies WRN inhibitor-sensitive MSI COAD/READ cell lines. **A-B** *WRN* (A) and *KMT2D* (B) KO lethality probabilities in *KMT2D* (A) and *WRN* (B) WT and LOF cancer cell lines. Cell lines were separated by microsatellite status as determined in Ghandi et al. [32]. ANOVA followed by Tukey’s HSD *p*-value * < 0.05, ** < 0.01, *** < 0.001, NS > 0.05. NA indicates cancer cell lines without MSI/MSS status annotations. **C** [AT]_n_ microsatellite repeat expansion size estimated by ExpansionHunter Denovo in six DepMap *KMT2D*^LOF^ cell lines from panel A that had WGS data and three *KMT2D*^WT+^ lines. WT+ denotes both wildtype alleles and missense mutations. Dashed lines indicate the average [AT]_n_ microsatellite repeat expansion size across all events. **D** *KMT2D* and MMR gene mutational status were annotated using DepMap data for MSI COAD/READ cell lines treated with the WRN inhibitors HRO761 and VVD-133214, described in Ferretti et al. [16] and Baltgalvis et al. [17], respectively. Cancer cell line names in red font indicate cell lines that did not have DepMap mutation information to verify mutational status

Given that *WRN* is a known essential gene in MSI cancer cell lines [8, 60, 62, 63, 122], we investigated whether microsatellite status played a role in the SL interaction predicted between *WRN* and *KMT2D*. To this end, we used MSI status to stratify the *WRN* KO lethality probability scores of *KMT2D*^LOF^ cell lines and *KMT2D*^WT^ cell lines from the pan-cancer dataset. We show that lethality probabilities in *KMT2D*^LOF^ MSI cell lines, resulting from *WRN* KO, were statistically significant and higher than *KMT2D*^WT^ cell lines, including the *KMT2D*^WT^ MSI group (ANOVA followed by Tukey’s HSD tests *p*-value < 0.001; Fig. 6A; Methods). We also found that *KMT2D*^LOF^ MSI lines had significantly higher *WRN* KO lethality probabilities than *KMT2D*^LOF^ microsatellite stable (MSS) cell lines (ANOVA followed by Tukey’s HSD test *p*-value < 0.001). However, no difference in *WRN* KO lethality probabilities was observed between *KMT2D*^WT^ MSI and *KMT2D*^WT^ MSS cell lines. This result indicated that *WRN* KO was specifically lethal in MSI cell lines harbouring *KMT2D*^LOF^ alterations. This analysis was not performed in a COAD/READ-specific manner due to a lack of COAD/READ cell lines in DepMap. Next, we used GRETTA to assess whether cancer cell lines with *WRN* LOF (*WRN*^LOF^) alterations might require *KMT2D* for survival. We found that *WRN*^LOF^ MSS lines were significantly more susceptible to *KMT2D* perturbation than *WRN* WT (*WRN*^WT^) MSS lines (ANOVA followed by Tukey’s HSD test *p*-value < 0.01; Fig. 6B; Additional file 15: Table S11; Methods). Our results are consistent with the notion that *WRN* is a SL interactor of *KMT2D* and that MSI alone may not confer a dependency on *WRN* for survival. In other words, our analysis indicated that *WRN* may be required for MSI cancer cell survival only when *KMT2D*^LOF^ mutations are present and that MSI alone may not result in *WRN* dependence, in contrast to initial descriptions [8, 60, 62, 63, 122].

Defects in MMR functions as a result of LOF mutations or epigenetic silencing of MMR-associated genes (including *MLH1*, *MLH3*, *MSH2*, *MSH3*, *MSH6*, and *PMS2*) are causes of MSI, resulting in numerous unrepaired mutations across the genome, mainly in repetitive sequences [161]. Although several studies have shown that *WRN* is a SL target of MSI cancer cells, restoration of MMR functions through ectopic expression of MMR-associated genes in MSI cell lines only showed partial rescue of cell viability [60] upon *WRN* KO, and generating MSI conditions through MMR gene KOs in MSS cell lines failed to recapitulate the lethal effects of *WRN* KO [122]. Therefore, we wanted to see whether *KMT2D*^LOF^ alterations in MSI cell lines would lead to a dependency on WRN for survival. To this end, we identified cancer cell lines, in which *WRN* KO was performed and the cell’s viability was validated (i.e. viability assays were performed on cell lines using a single sgRNA targeting *WRN* instead of a pooled sgRNA KO screen) and analysed whether MSI cell lines that harboured *KMT2D*^LOF^ alterations showed more consistent lethal effects upon *WRN* KO compared to *KMT2D*^WT^ MSI cell lines. We identified 32 cancer cell lines consisting of COAD/READ, STAD, UCEC, and ovarian (OV) cancers that had genomic sequencing data from DepMap and (in)dependence on WRN for survival validated by studies conducted by either Behan et al. [8], Chan et al. [60], Kategaya et al. [61], Lieb et al. [62], or Picco et al. [63] (Additional file 2: Table S2A). For these cell lines, we annotated the presence of LOF alterations in *KMT2D* and MMR genes, namely *EXO1*, *MBD4*, *MLH1*, *MSH2*, *MSH3*, *MSH5*, *MSH6*, *PMS1*, *PSM2*, and *RFC1* [63], reported MSI status, and the outcomes of *WRN* KO viability assays (Additional file 16: Fig. S5A; Methods; Additional file 2: Table S2B). Among the 14 cell lines that showed *WRN* was an essential gene (i.e. that required *WRN* for survival), we identified LOF alterations of MMR genes in 85% (12/14) and concurrent *KMT2D*^LOF^ alterations in 64% (9/14). Among the 18 cell lines where *WRN* was reported as a non-essential gene, we found only 33% with MMR or *KMT2D* alterations (4/18 with MMR gene LOF and 2/18 with *KMT2D*^LOF^). These results indicate that most MMR-deficient lines harbour *KMT2D*^LOF^ alterations and that not all MMR-deficient cell lines require WRN for survival. Furthermore, both *KMT2D*^LOF^ alterations and LOF alteration in MMR genes were significantly enriched in cell lines where *WRN* was essential (Fisher’s exact test *p*-value = 0.0007 with OR = 20 and *p*-value = 0.0009 with OR = 21, respectively; Additional file 16: Fig. S5B-D). However, LOF alterations in *KMT2D* and MMR also co-occurred (Fisher’s exact test *p*-values = 0.0091 and OR = 9; Additional file 16: Fig. S5D). Given the limited sample size and mixture of cancer types, we were not able to distinguish the independent effects of *KMT2D*^LOF^ alterations and MMR deficiency in specific cancer types.

Next, given that MSI cells require WRN to resolve expanded microsatellite regions containing [AT]_n_ repeat elements for survival and that these expansions could not be recapitulated by MSS cells upon MMR gene KO [122], we sought to determine whether the loss of *KMT2D* affected the expansion of [AT]_n_ microsatellite regions in MSI cell lines. To this end, we used ExpansionHunter Denovo [65] (Methods) to profile [AT]_n_ microsatellite regions in WGS data from DepMap cancer cell lines (SRA project: PRJNA523380; Additional file 17: Table S12A). Briefly, ExpansionHunter Denovo is a catalogue-free method for genome-wide repeat expansion detection and has been used previously to estimate [AT]_n_ repeat element expansion [122]. We characterised microsatellite regions of 14 cell lines, including nine *KMT2D*^LOF^ MSI, two *KMT2D*^WT^ MSI, and two *KMT2D*^WT^ MSS lines, and subsequently filtered for lines that contained more than one anchored in-repeat read (i.e. one read pair maps within and the other outside of a repeat region) in [AT]_n_ regions. This resulted in profiles of nine cell lines, specifically CCK81, LOVO, SW48, LS180, MFE319, RKO, SKMEL2, NCIH747, and COLO201 lines. Interestingly, between *KMT2D*^LOF^ MSI, *KMT2D*^WT^ MSI, and *KMT2D*^WT^ MSS lines, [AT]_n_ sites were estimated to be the most expanded in *KMT2D*^LOF^ MSI cell lines, namely LOVO, SW48, LS180 (COAD/READ), and MFE319 (UCEC; Fig. 6C; Additional file 17: Table S12B). The LOVO [61] and SW48 [8] lines were previously shown to depend on WRN for survival. Two *KMT2D*^LOF^ MSI lines, CCK81 and RKO (a line that also depends on WRN for survival) [62], showed below average [AT]_n_ expansion size across events (Fig. 6C; mean [AT]_n_ expansion size across samples = 193); however, the number of [AT]_n_ regions detected was among the highest of all samples in both lines. Two *KMT2D*^WT^ lines, SKMEL2 (MSI) and COLO201 (MSS), also showed below-average [AT]_n_ expansion size. The *KMT2D*^WT^ MSS cell line NCIH747 showed above-average [AT]_n_ expansion size; however, the [AT]_n_ size was > 50% below that of the expanded *KMT2D*^LOF^ MSI lines. Although we were not able to make robust statistical comparisons of [AT]_n_ expansion between *KMT2D*^WT^ MSI and *KMT2D*^LOF^ MSI cell lines due to lack of available public domain data for MSI cell lines, our results showed a pattern consistent with the notion that *KMT2D*^LOF^ alteration is associated with increased size and number of expanded [AT]_n_ microsatellite repeat regions.

While this manuscript was in preparation, Ferretti et al. [16] and Baltgalvis et al. [17] independently reported WRN inhibitors, HRO761 and VVD-133214, respectively, which are in phase I clinical trials (NCT05838768 and NCT06004245). Both phase I trials are recruiting patients with advanced metastatic solid tumours that are MSI or MMR gene-deficient. Interestingly, both papers reported MSI COAD/READ cell lines that were insensitive to WRN inhibitors [16, 17]. We sought to determine whether cell line sensitivity to these WRN inhibitors could be explained by *KMT2D* mutational status. Using DepMap data, we annotated the *KMT2D* and MMR gene mutational statuses of the MSI COAD/READ cell lines treated with HRO761 or VVD-133214 (Methods), and compared these annotations to the inhibitor sensitivity data reported for the cell lines in [16] and [17]. With the exception of the KM12L4 and LS174T lines, which were WRN inhibitor-sensitive but did not have DepMap mutational data available for our analysis, we were able to show that *KMT2D*^LOF^ alterations were present in all drug-sensitive cell lines (Fisher’s exact test *p*-value = 0.0035; Fig. 6D; Table 3). Interestingly, KM12L4 and LS174T originate from the same primary COAD/READ patient biopsy sample as the *KMT2D*^LOF^ MSI cell lines KM12 [162] and LS180 [163, 164], respectively (shown in Fig. 6D and Additional file 16: Fig. S5A). The KM12L4 cell line was established from spontaneous metastatic cells that were produced from the KM12 cell line being injected into the mouse spleen [162]. The LS174T and LS180 cell lines were derived from the same primary culture, where LS174T and LS180 lines were established from a trypsinized and a scrapped non-trypsinized passage, respectively [163]. It is thus entirely possible that the KM12L4 and LS174T cell lines harbour the same *KMT2D*^LOF^ alterations as KM12 and LS180. However, even without mutational data for the KM12L4 and LS174T cell lines, our results significantly associate sensitivity to WRN inhibitors of MSI COAD/READ cell lines with *KMT2D*^LOF^ mutations as we predicted using in silico GI mapping. Thus, using in silico GI mapping predictions and in vitro experimental data, we now show that *KMT2D*^LOF^ mutations are able to identify MSI COAD/READ cell lines that are sensitive to WRN perturbation.

**Table 3:** A contingency table comparing the frequency of *KMT2D*^LOF^ alterations and WRN inhibitor-treatment sensitivity in MSI COAD/READ cell lines in Fig. 6D. WT + Mis denotes both wildtype allele and missense mutations. KM12L4 and LS174T cell lines were excluded from this analysis due to a lack of KMT2D status information

		WRN inhibitor sensitivity	
		Sensitive	Insensitive
Alteration (SNV + CNV)	*KMT2D*^LOF^	10	0
	*KMT2D*^WT+Mis^	0	3

Fisher’s exact text *p *- value = 0.022; OR = Inf

## Discussion

In this study, we demonstrated a framework for revealing tumour suppressor gene functions and vulnerabilities of cancer cells harbouring LOF alterations in them. Using the tumour suppressor gene *KMT2D* as an example for our framework, we performed in silico genetic network analyses to gain further insights into its functions and identify its cancer type-specific genetic interactors. We identified genes in *KMT2D*’s essentiality network and proteins in its proteomic network that were known interactors and also new candidate interactors that have roles associated with the regulation of cell cycle, cell division, telomere maintenance, chromosome segregation, DNA replication and metabolism, thus expanding the extent of KMT2D’s role in these processes. We identified SL and AL candidates that have roles in mitotic processes, metabolic processes, and immune response. Furthermore, results from the in silico GI screens identified several SL candidates—namely *NDUFB5* (in the STAD screen), *MDM2* and *TUBA1B* (in the COAD/READ screen), and *WRN* (in the pan-cancer and COAD/READ screens)—which encode proteins that can be inhibited using existing and in-development drugs. Using TCGA-STAD cases, we showed that *NDUFB5* expression was elevated, and glycolytic genes were significantly elevated in *KMT2D*^LOF^ cases. Using TCGA-COAD/READ cases, we showed that the mRNA expression of *MDM2*, a p53 inhibitor, was significantly increased, and the expression of p53 downstream targets was dysregulated in *KMT2D*^LOF^ cases. Interestingly, in addition to showing genes associated with immune response as *KMT2D* SL interactors, which included *TUBA1B*, we also showed that ICI response makers were elevated in *KMT2D*^LOF^ cases. Using untreated TCGA-COAD/READ cases, we showed that *KMT2D*^LOF^ MSI cases have a trending increase in *TUBA1B*,* CD274*, and *CTLA4* mRNA expression, CD8 + T-cell infiltration scores, and favourable ICI treatment response scores and have significantly higher TMB, neoantigen scores, cytolytic activity, IFNγ signalling scores, and γδ T-cell infiltration scores compared to *KMT2D*^WT^ cases. In our retrospective analysis of heavily treated POG cohorts, we found similar trends as in TCGA cohorts. Finally, we also showed that KMT2D may mediate MSI cell line dependency on WRN for survival, possibly through further expansion of [TA]_n_ microsatellite regions. We also showed that *KMT2D* mutational status was significantly associated with WRN inhibitor sensitivity in MSI cell lines. Our study supported and expanded previously published KMT2D functions in regulating genomic stability [72, 89], metabolism [117, 118], and immune response [165–168]; presented promising SL interactors that can potentially be targeted using drugs, including two in clinical trials; and proposes ICIs and/or WRN inhibitors as potential therapeutic avenues for *KMT2D*^LOF^ MSI cases.

Although KMT2D is best characterised for its role as a histone methyltransferase, loss of *KMT2D* has been associated with increased genomic instability, dysregulation of DNA repair/replication [72, 89], and metabolic dysregulation [117, 118]. However, the extent of KMT2D’s involvement in regulating genomic stability and metabolism is still unclear. In addition to supporting KMT2D’s known role as a histone modifier, our analysis of *KMT2D*’s co-essential network and chromatin-specific proteome interaction network revealed an enrichment of genes/proteins related to cell cycle processes, mitotic segregation, and DNA replication/repair that have not previously been linked to KMT2D. These novel candidate SL interactors were also involved in mitotic regulation and homologous recombination. These processes are known to affect genomic stability [169] and thus further implicate KMT2D in this role.

For the first time, we predicted *KMT2D* candidate SL interactors, namely *NDUFB5*, *MDM2*, *TUBA1B*, and *WRN*, which encode targets of existing drugs or those that are in development. We identified regulators of the OXPHOS pathway, namely *NDUFB5* [115] (a priority class A SL candidate) and *OGT* [170] (a candidate co-essential gene and its encoded protein was detected in the ChIP-MS analysis), and showed that glycolytic genes were dysregulated in *KMT2D*^LOF^ TCGA-STAD cases. These results showing dysregulation of metabolic processes were consistent with the studies conducted in KMT2D-deficient mouse lung adenocarcinoma cell lines [117] and in Kabuki patient-derived cells [118]. Interestingly, the OXPHOS pathway (NDUFB5 is a component of complex I), glycolysis, and OGT are known to contribute to the de novo pyrimidine synthesis [171, 172], a pathway that is garnering increasing attention as a therapeutic target [173]. A recent study by Gwynne et al. [172] has shown that, in medulloblastoma, MYC (a downstream target of the MDM2/p53 pathway [138]) is stabilised by O-GlcNAcylation and that medulloblastoma cells were sensitive to inhibition of the de novo pyrimidine synthesis. Notably, our analyses also identified *MDM2* as a priority class A SL candidate and that *MDM2* and p53 downstream targets were dysregulated in *KMT2D*^LOF^ TCGA-COAD/READ cases. Taken together, we posit that *KMT2D*^LOF^ cases may also be more sensitive to de novo pyrimidine synthesis inhibitors than *KMT2D*^WT^ cases and that further studies are warranted to determine whether *KMT2D* mutations may be a biomarker for such targeted therapies.

We also contributed results that further implicate KMT2D with a role in immune response regulation. MSI is a marker for ICI treatment; however, only ~ 53% of MSI cases have an objective response to ICI treatment [174], suggesting additional biomarkers are necessary to stratify MSI cases. Several biomarkers have been proposed for ICI treatment, including HRD [175], *ARID1A*^LOF^ [176], and *SETD2*^LOF^ [177]. Wang et al. [165] also demonstrated that mice transplanted with KMT2D-deficient tumours were responsive to ICI treatment and that TMB was elevated in TCGA cases with *KMT2D*^LOF^. In this study, we showed elevated TMB in the MSS *KMT2D*^LOF^ cases compared to the MSS *KMT2D*^WT^ in the POG and MSK-IMPACT COAD/READ cohorts, consistent with previous studies showing loss of KMT2D leading to increased genomic instability [72, 89]. We showed for the first time that candidate *KMT2D* SL interactors were involved in T-cell cytotoxicity and immune response regulation. Notably, we identified *TUBA1B* as a highly attractive SL candidate, where its overexpression has recently been implicated in favourable ICI treatment outcomes [111]. We not only showed that *TUBA1B* was overexpressed in *KMT2D*^LOF^ MSI cancers compared to *KMT2D*^WT^ MSI cancers in two cohorts (TCGA and POG cohorts), but we also showed that several established ICI response markers were elevated in these cases. Although our analyses of TCGA, POG, and MSI-IMPACT cohorts had a small number of *KMT2D*^LOF^ MSI cases, our data were compatible with the notion that *KMT2D*^LOF^ MSI cases might be more sensitive to ICI treatment than *KMT2D*^WT^ MSI cases. Our data were consistent with previous studies, which also used small cohorts, to show that a larger proportion of *KMT2D*^LOF^ cases had a more durable response to ICI treatment than *KMT2D*^WT^ cases [165, 178]. However, given that large ICI-treated cohort studies, such as KEYNOTE-177 [NCT02563002], do not collect genomic data, there is an unmet future need for cohorts with sufficient sample sizes of *KMT2D*^WT^ MSI cases and *KMT2D*^LOF^ MSI cases to perform more robust statistical analyses. Altogether, these results suggest that *KMT2D*^LOF^ MSI cancers may respond to ICI treatments, and we thus propose *KMT2D* as a possible biomarker to further stratify MSI cases for ICI treatment.

Finally, the SL interaction between *KMT2D* and *WRN* contributes a new layer of understanding in MSI cancer cell vulnerability and potential strategies for identifying patients sensitive to WRN inhibitors. We showed for the first time that MSI cell lines harbouring *KMT2D*^LOF^ alterations were more sensitive to *WRN* KO than *KMT2D*^WT^ MSI cell lines, contrary to the previous studies indicating that the MSI feature alone is sufficient for cancer cells to confer a survival vulnerability to WRN perturbation [8, 60, 62, 63, 122]. Although it is unclear how KMT2D may directly mediate survival dependency on WRN in MSI cancer cells, it is interesting that *MSH6*, a gene that functions in MMR [98], appeared as a co-essential gene and protein interactor of KMT2D. We also showed that [AT]_n_ repeat elements were relatively larger and more abundant than *KMT2D*^WT^ MSI cell lines. Further studies are needed to better understand the possible mechanisms of the KMT2D-MMR relationship. Most notably, we used the *KMT2D*^LOF^ mutational status to identify MSI cancer cell lines sensitive to WRN inhibitors, which are in phase I clinical trials. Given the lack of WRN inhibitor response data in cancer patients harbouring *KMT2D*^WT^ and *KMT2D*^LOF^ tumours, the lack of a relationship between *KMT2D* status and WRN inhibitors in patient samples (e.g. biopsies) remains a limitation of our study. This limitation could, in principle, be addressed in a future study, perhaps associated with a clinical trial assessing drug efficacy, that seeks to determine whether a patient’s *KMT2D* mutational status correlates with response to WRN inhibitors. Furthermore, given that ICI-resistant MSI xenograft models have been shown to be sensitive to the VVD-133214 WRN inhibitor [17], we speculate that ICI-refractory patients with *KMT2D*^LOF^ mutations may respond to WRN inhibitors.

## Conclusions

Our study of in silico genetic networks identified several potential roles for KMT2D and further implicated it in regulating genomic stability and metabolism. We highlighted cancer type-specific genetic interactors, namely *NDUFB5*, *MDM2*, *TUBA1B*, and *WRN*, which were promising targets of existing and in-development drugs. Furthermore, we used cancer patient data to provide evidence for *KMT2D*^LOF^ alterations as a potential biomarker for ICI and WRN inhibitor treatments. Altogether, our work serves as an example for identifying novel functional associations and potential targeted treatment opportunities for cancers with tumour suppressor gene LOF alterations.

## Supplementary Information

Additional file 1: Table S1. TCGA cancer type assigned to DepMap disease types.

Additional file 2: Table S2. WRN KO and WRN inhibitor treatment validated cell lines. A. List of DepMap cell lines previously for WRN survival dependence using WRN KO assays. DepMap_ID: DepMap cell line id. Cell_line_name: Name of cell line. Author: name of publication WRN KO assay was performed in. WRN_dependence: outcome of WRN KO (TRUE indicates that WRN was essential for survival and FALSE indicates that it was not). B. Mutations found in MMR genes and KMT2D in cell lines in A. C-D. Shows a list of DepMap cell lines (C) and their MMR gene and KMT2D (D) mutations treated with WRN inhibitors.

Additional file 3: Table S3. KMT2D essentiality network results. A. Ranked list of genes in the KMT2D essentiality network. GeneName: hugo gene symbol. GeneNameID: hugo gene symbol separated by NCBI gene id. Estimate: Pearson’s correlation coefficient. *P*.value: *p*-value derived from Pearson’s correlation test. Perm.adj.*p*.value: *p*-values corrected for multiple testing using permutation tests with 100,000 random sampling. Rank: rank in list determined by order of estimate. Candidate_inflection: TRUE if a gene’s Pearson’s correlation coefficient is past the inflection point of the positive/negative curve (see Methods). Chr: chromosome location of gene. Gene_start_bp: a gene’s start position. Gene_end_bp: a gene’s end position. Karyotype_band: the karyotype band in which the gene is found. B. Enriched GO biological processes found among co-/anti-essential genes. Results are outputs from ClusterProfiler. C. Clustered GO terms of co-essential genes.

Additional file 4: Table S4. KMT2D ChIP-MS results. A. KMT2D ChIP-MS detected proteins. Accession: uniprot id. Gene: hugo gene symbol. LogFC_HL_kmt2d_R1KO1: log10 fold change (heavy isotope/light isotope) of replicate 1 of HEK-KMT2DKO1. LogFC_HL_kmt2d_R2KO1: log10 fold change (heavy isotope/light isotope) of replicate 2 of HEK-KMT2DKO1. LogFC_HL_kmt2d_R1KO3: log10 fold change (heavy isotope/light isotope) of replicate 1 of HEK-KMT2DKO3. LogFC_HL_kmt2d_R2KO3: log10 fold change (heavy isotope/light isotope) of replicate 2 of HEK-KMT2DKO3. Num_reps_with_peptides: number of replicates with peptides detected. B. Enriched GO biological processes found among detected proteins. Results are outputs from ClusterProfiler. C. Clustered GO terms. D. A list of KMT2D interactors that overlap with KMT2D co-/anti-essential genes.

Additional file 5: Fig. S1. Characterisation of KMT2D mutations, expression, and global histone levels in DepMap cancer cell lines. A. Lollipop plots showing SNVs and small insertions and deletions in KMT2D identified in DepMap cell lines by cancer type. B. KMT2D mRNA expression in transcript per million (TPM) for KMT2DWT and KMT2DLOF DepMap cancer cell lines datasets. C. Relative concentration of global histone marks across cancer types. Benjamini Hochberg (BH)-corrected Welch’s t-test *p*-values † < 0.1, * < 0.05, ** < 0.01, *** < 0.001 and NS > 0.1. NA indicates comparisons that are not analysed due to small sample size (N < 3).

Additional file 6: Table S5. Results GI predictions using GRETTA. A. List of DepMap cancer cell lines by KMT2DWT/KMT2DLOF genotypes and cancer type. DepMap_ID: DepMap cancer cell lines IDs. stripped_cell_line_name: cell line names provided by DepMap without special characters or spaces. disease: cancer type assigned by DepMap. disease_subtype: cancer subtype assigned by Depmap. TCGA_type: matched TCGA cancer type abbreviation (see Methods for details). Total_mutations: total number of mutations detected (LOF and non-LOF). LOF_mutations: number of LOF SNVs and insertion deletions detected (see Methods). CN_status: copy number status. Group: KMT2D group assigned (Control refers to the KMT2DWT group). B. All KMT2D GI prediction results by cancer context. Cancer type context: cancer context KMT2D GI screens were performed in. GeneNameID: hugo gene name with Entrez gene ID. GeneNames: gene name without Entrez gene ID. Control_median: median lethality probability of KMT2DWT cell line group. Mutant_median: median of KMT2DLOF group lethality probabilities. Control_sd: standard deviation of KMT2DWT lethality probabilities. Mutant_sd: standard deviation of KMT2DLOF lethality probabilities. Pval: uncorrected Mann Whitney U-test *p*-values comparing KMT2DWT and KMT2DWT lethality probabilities. log2FC_by_median: log2-transformed fold change of KMT2DLOF over KMT2DWT lethality probabilities. Interaction_score: GI interaction score for visualisation (see Methods). Adj_pval: Mann Whitney U-test *p*-values adjusted for multiple testing using permutation test with 10,000 random samplings. GI_direction: whether a GI is alleviating (AL) or synthetic lethal (SL). C. Prioritisation classification assigned to significant GIs (GI prediction tiers I, II, and III). First 17 column names are the same as A. Drug group: drug tractability group assigned to GI. Priority_class: priority class assigned to GI.

Additional file 7: Table S6. Expression of NDUFB5, PER2, and PER2-downstream targets in TCGA-STAD cases. A. TCGA-STAD cohort NDUFB5, PER2, and PER2-target gene expression. tpm: transcript per million (see Methods section).

Additional file 8: Table S7. Expression of MDM2, TP53, and TP53-downstream targets in TCGA-COAD/READ cases. A. TCGA-COAD/READ cohort KMT2D and TP53 mutations and expression of MDM2, TP53, and TP53-downstream targets. mut: WT, LOF, or Other mutation called. cn: copy number status, tpm: transcript per million.

Additional file 9: Table S8. Profiles of KMT2D mutations and immune markers in TCGA-COAD/READ cases. A. TCGA-COAD/READ clinical data and immune marker profiles. OS: overall survival. Fpkm: fragments per kilobase of transcript per million mapped reads. B_cells_naive to absolute_score_sig_score columns are immune cell composition score outputs from Cibersort. PredictIO_score: immune checkpoint response score (see Methods).

Additional file 10: Table S9. Age and mutational signature activity scores of TCGA-COAD/READ cases.

Additional file 11: Fig. S2. Mutational signatures and Cibersort immune composition of TCGA-COAD/READ cohorts. A. A comparison of single base pair substitution (SBS) mutational signatures. Uncorrected Welch’s t-test *p*-values * < 0.05 and ** < 0.01, and not analysed (NA) when sample size was < 3. Not significant after multiple-testing correction (BH-corrected *p*-value > 0.05). B. A comparison of chronological age. Welch’s t-test *p*-values NS > 0.1 and not analysed (NA) when sample size was < 3. C. Distribution of relative cell type fractions (left) and a comparison of absolute cell type fractions (right) calculated by Cibersortx in TCGA MSI COAD/READ cohort. Relative cell type fractions are shown in the left panels. BH-corrected Welch’s t-test *p*-values † < 0.1, * < 0.05, ** < 0.01, *** < 0.001, and NS > 0.1.

Additional file 12: Table S10. Profiles of KMT2D mutations and immune markers in POG and MSK-IMPACT cases. A. POG solid cancer clinical data and immune marker profiles. OS: overall survival. censor_status: 1-living, 2-deceased. Fpkm: fragments per kilobase of transcript per million mapped reads. B_cells_naive to absolute_score_sig_score columns are immune cell composition score outputs from Cibersort. PredictIO_score: immune checkpoint response score (see Methods section). B. MSK-IMPACT cohort overall survival and TMB obtained from Samstein et al. (13), and MSI and KMT2D status (see Methods section).

Additional file 13: Fig. S3. Overall survival and TMB of MSK-IMPACT cohorts. A. Proportion of advanced and metastatic MSI-IMPACT COAD/READ (left) and all solid cancer (right) cases with MSI and KMT2DLOF alterations. B. Number of cases by cancer type found in the MSK-IMPACT solid cancer cohort in panel A. C. Kaplan–Meier curves (top) and risk table (bottom) comparing overall survival of KMT2DLOF cases to KMT2DWT cases. Groups with less than 10 cases were not analysed (NA). D. A comparison of TMB between KMT2DWT and KMT2DLOF MSI/MSS cases in MSK-IMPACT COAD/READ and solid cancer cohorts. Welch’s t-test *p*-value * < 0.05 and *** < 0.001. Groups that did not reach 0.5 overall survival probability (C) or with less than three cases (D) were not analysed (NA).

Additional file 14: Fig. S4. Cibersort immune composition of POG cohorts. A-C. Distribution of relative cell type fractions (left) and a comparison of absolute cell type fractions (right) calculated by Cibersortx in POG MSI-COAD/READ (A), MSS-solid cancer (B), and MSI-solid cancer (C) cohorts. Relative cell type fractions are shown in the left panels. BH-corrected Welch’s t-test *p*-values † < 0.1, * < 0.05, and NS > 0.05.

Additional file 15: Table S11. List of DepMap cancer cell lines in WRNWT/WRNLOF groups. A. DepMap_ID: DepMap cancer cell lines IDs. stripped_cell_line_name: cell line names provided by DepMap without special characters or spaces. disease: cancer type assigned by DepMap. disease_subtype: cancer subtype assigned by Depmap. MS_status: inferred microsatellite status by Ghandi et al. (2019). Group: WRN group assigned (Control refers to the WTWT group; pan-cancer). Total_mutations: total number of mutations detected (LOF and non-LOF). LOF_mutations: number of LOF SNVs and insertion deletions detected (see Methods). CN_status: copy number status. KMT2D_prob: lethality probability when KMT2D is knocked out in the cell line. B. DepMap cancer cell lines that were previously validated to be dependent or independent of WRN. DepMap_ID: DepMap cancer cell lines IDs. stripped_cell_line_name: Cell line names provided by DepMap without special characters or spaces. disease: cancer type assigned by DepMap. Author: first author of study. WRN_dependence: whether authors showed WRN dependence. TRUE indicates KO of WRN was lethal to cell lines and FALSE indicates that cells were viable when WRN was knocked out. C. KMT2D and MMR mutations detected by DepMap WGS/WES characterization in cell lines from B.

Additional file 16: Fig. S5. MSI cancer cells that require WRN for survival frequently harbour KMT2DLOF mutations. Proportion of advanced and metastatic MSI-IMPACT COAD/READ (left) and all solid cancer (right) cases with MSI and KMT2DLOF alterations. B. Number of cases by cancer type found in the MSK-IMPACT solid cancer cohort (panel A). C. Kaplan–Meier curves (top) and risk table (bottom) comparing overall survival of KMT2DLOF cases to KMT2DWT cases. D. A comparison of TMB between KMT2DWT and KMT2DLOF MSI/MSS cases in MSK-IMPACT COAD/READ and solid cancer cohorts. Welch’s t-test *p*-value * < 0.05 and *** < 0.001. Groups of less than 3 were not analysed (NA).

Additional file 17: Table S12. Characterising [AT]n motif expansion in cancer cell lines. A. All MSI DepMap cancer cell lines with WGS data and three MSS DepMap cell lines with WGS data for comparison. Cell_line_stripped_name: cell line names provided by DepMap without special characters or spaces. disease: cancer type assigned by DepMap. MSI_status: inferred microsatellite status by Ghandi et al. (2019). KMT2D_group: KMT2D group assigned to a cell line. SRA_project_ID: NCBI SRA project ID for raw WGS data. SRA_Run_ID: NCBI SRA run ID for raw WGS data. Instrument: sequencer used to perform WGS. B. Profiles of [AT]n motifs generated using ExpansionHunter Denovo. Cell_line_name: cell line names provided by DepMap without special characters or spaces. disease: cancer type assigned by DepMap. SRA_id: NCBI SRAN run ID. group: MSI status and KMT2D group of cell line. Contig: chromosome location of motif. Start: start site (bp) of motif. End: end site (bp) of motif. Motif: motif type. Num_anc_irrs: number of anchored in-repeat reads (i.e. one read pair maps within and the other outside of a repeat region; see Methods). norm_num_anc_irrs: normalised number of anchored in-repeat reads. Het_str_size: estimated motif repeat size.

## Data Availability

The scripts for reproducing the analysis, figures, and tables are available on GitHub (https://github.com/ytakemon/KMT2D_genetic_network_study/; [179]). The GRETTA R package and DepMap data version 20Q1 [31] used in this study are publicly available on the GRETTA GitHub repository (https://github.com/ytakemon/GRETTA/; [30, 67]). GRETTA has been archived with a citable DOI on Zenodo (10.5281/zenodo.6940757/) and a Singularity container has been made available on Sylabs (https://cloud.sylabs.io/library/ytakemon/gretta/gretta/). ChIP-MS data have been deposited with the ProteomeXchange Consortium via the Proteomics Identification (PRIDE [180]) database (accession PXD048272; https://www.ebi.ac.uk/pride/archive/projects/PXD048272/; [93]). Genomic and transcriptomic sequence datasets pertaining to the POG cohort, including metadata with library construction and sequencing approaches have been deposited at the European Genome–phenome Archive [181] as part of the EGAS00001001159 study with accession numbers as listed in Additional file 12: Table S10. All other relevant data supporting the key findings of this study are available in the Additional files section.
