# Supplementary figures and images for: Mapping in silico genetic networks of the KMT2D tumour suppressor gene to uncover novel functional associations and cancer cell vulnerabilities
Source: Genome Med. 2024 Nov 22;16:136. doi: 10.1186/s13073-024-01401-9 (PMC11583415; doi:10.1186/s13073-024-01401-9)

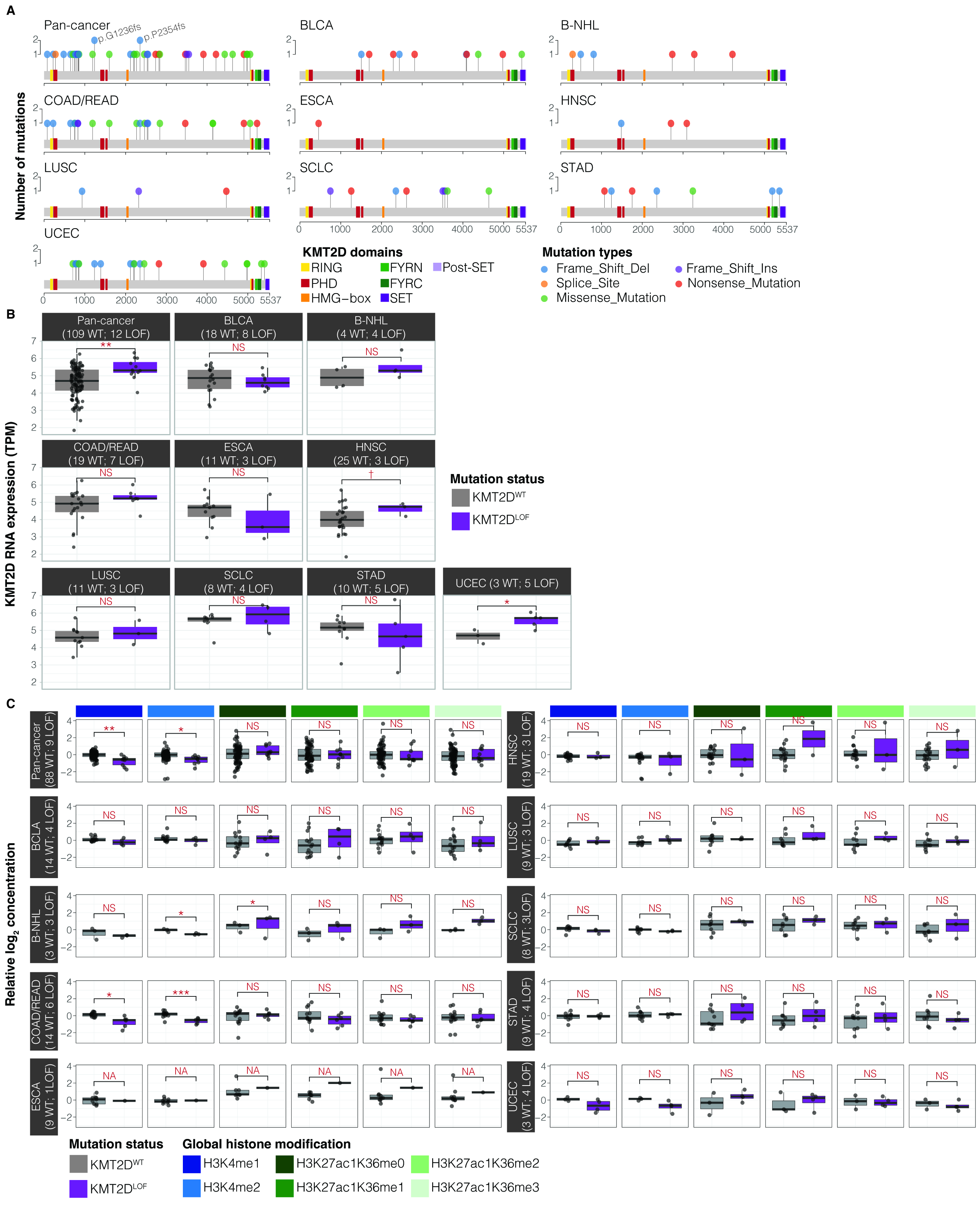

Supplement: Supplementary file 5 — Additional file 5: Fig. S1. Characterisation of KMT2D mutations, expression, and global histone levels in DepMap cancer cell lines. A. Lollipop plots showing SNVs and small insertions and deletions in KMT2D identified in DepMap cell lines by cancer type. B. KMT2D mRNA expression in transcript per million (TPM) for KMT2DWT and KMT2DLOF DepMap cancer cell lines datasets. C. Relative concentration of global histone marks across cancer types. Benjamini Hochberg (BH)-corrected Welch’s t-test p-values † < 0.1, * < 0.05, ** < 0.01, *** < 0.001 and NS > 0.1. NA indicates comparisons that are not analysed due to small sample size (N < 3). [file 13073_2024_1401_MOESM5_ESM.tif]

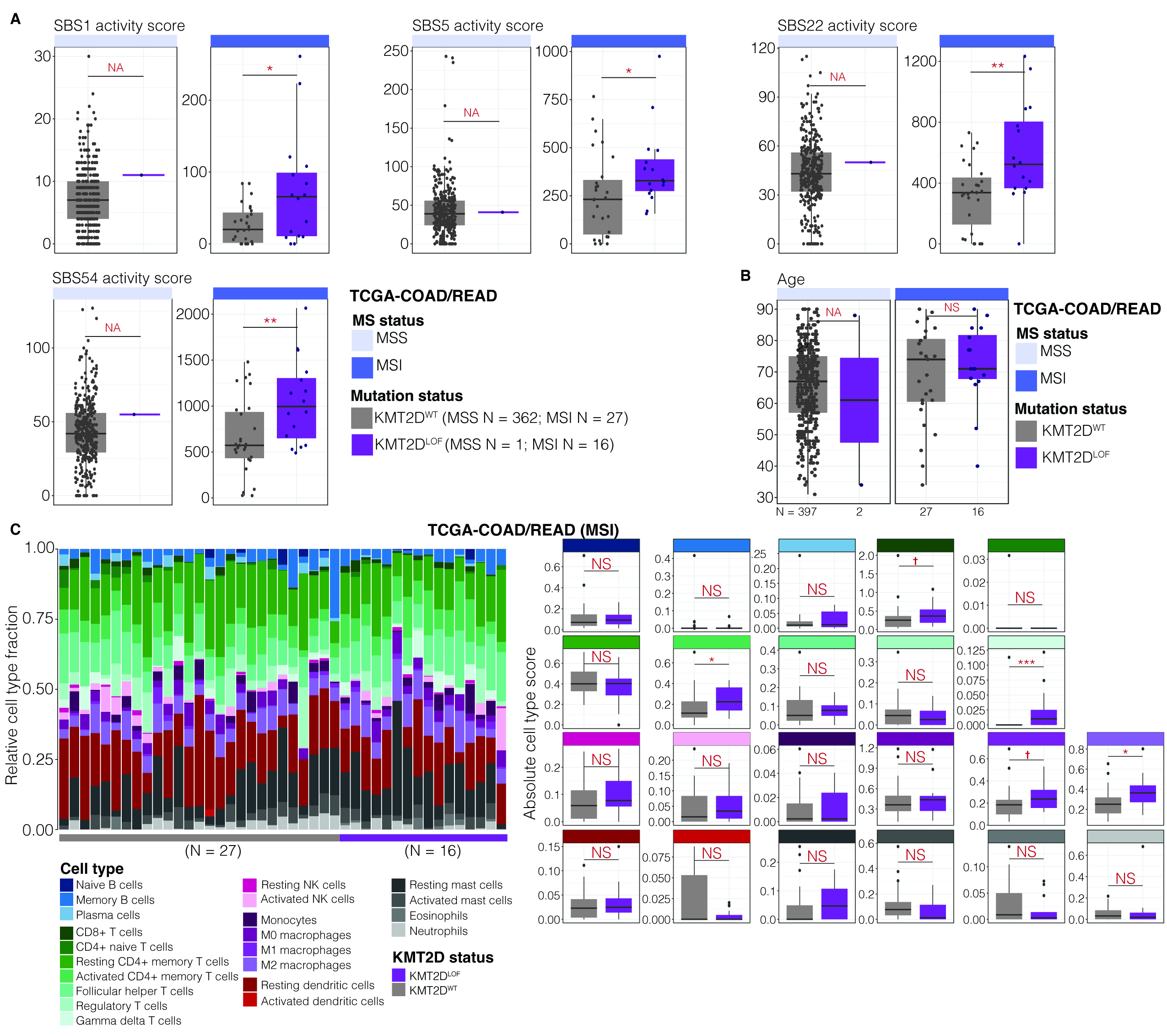

Supplement: Supplementary file 11 — Additional file 11: Fig. S2. Mutational signatures and Cibersort immune composition of TCGA-COAD/READ cohorts. A. A comparison of single base pair substitution (SBS) mutational signatures. Uncorrected Welch’s t-test p-values * < 0.05 and ** < 0.01, and not analysed (NA) when sample size was < 3. Not significant after multiple-testing correction (BH-corrected p-value > 0.05). B. A comparison of chronological age. Welch’s t-test p-values NS > 0.1 and not analysed (NA) when sample size was < 3. C. Distribution of relative cell type fractions (left) and a comparison of absolute cell type fractions (right) calculated by Cibersortx in TCGA MSI COAD/READ cohort. Relative cell type fractions are shown in the left panels. BH-corrected Welch’s t-test p-values † < 0.1, * < 0.05, ** < 0.01, *** < 0.001, and NS > 0.1. [file 13073_2024_1401_MOESM11_ESM.tif]

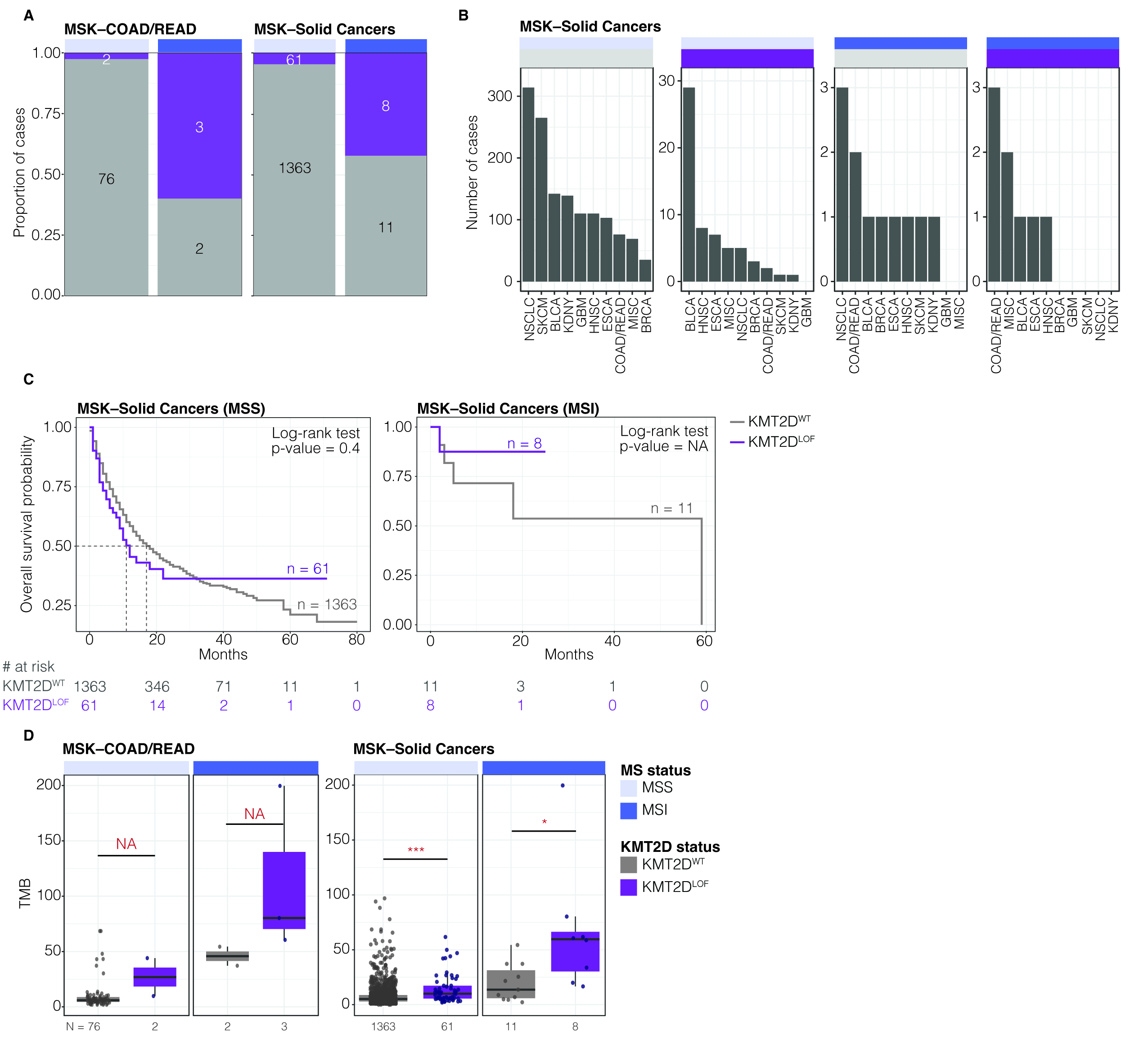

Supplement: Supplementary file 13 — Additional file 13: Fig. S3. Overall survival and TMB of MSK-IMPACT cohorts. A. Proportion of advanced and metastatic MSI-IMPACT COAD/READ (left) and all solid cancer (right) cases with MSI and KMT2DLOF alterations. B. Number of cases by cancer type found in the MSK-IMPACT solid cancer cohort in panel A. C. Kaplan–Meier curves (top) and risk table (bottom) comparing overall survival of KMT2DLOF cases to KMT2DWT cases. Groups with less than 10 cases were not analysed (NA). D. A comparison of TMB between KMT2DWT and KMT2DLOF MSI/MSS cases in MSK-IMPACT COAD/READ and solid cancer cohorts. Welch’s t-test p-value * < 0.05 and *** < 0.001. Groups that did not reach 0.5 overall survival probability (C) or with less than three cases (D) were not analysed (NA). [file 13073_2024_1401_MOESM13_ESM.tif]

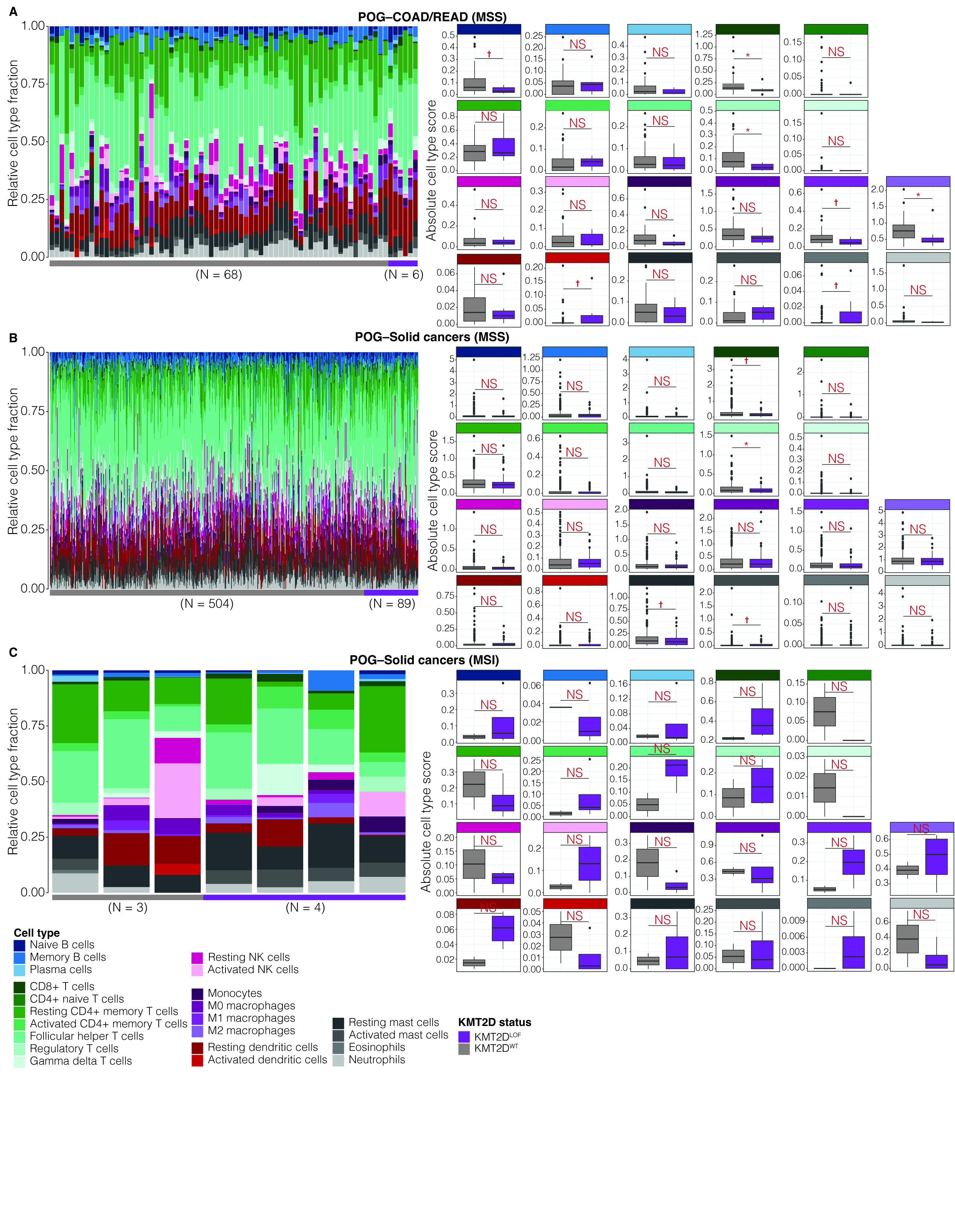

Supplement: Supplementary file 14 — Additional file 14: Fig. S4. Cibersort immune composition of POG cohorts. A-C. Distribution of relative cell type fractions (left) and a comparison of absolute cell type fractions (right) calculated by Cibersortx in POG MSI-COAD/READ (A), MSS-solid cancer (B), and MSI-solid cancer (C) cohorts. Relative cell type fractions are shown in the left panels. BH-corrected Welch’s t-test p-values † < 0.1, * < 0.05, and NS > 0.05. [file 13073_2024_1401_MOESM14_ESM.tif]

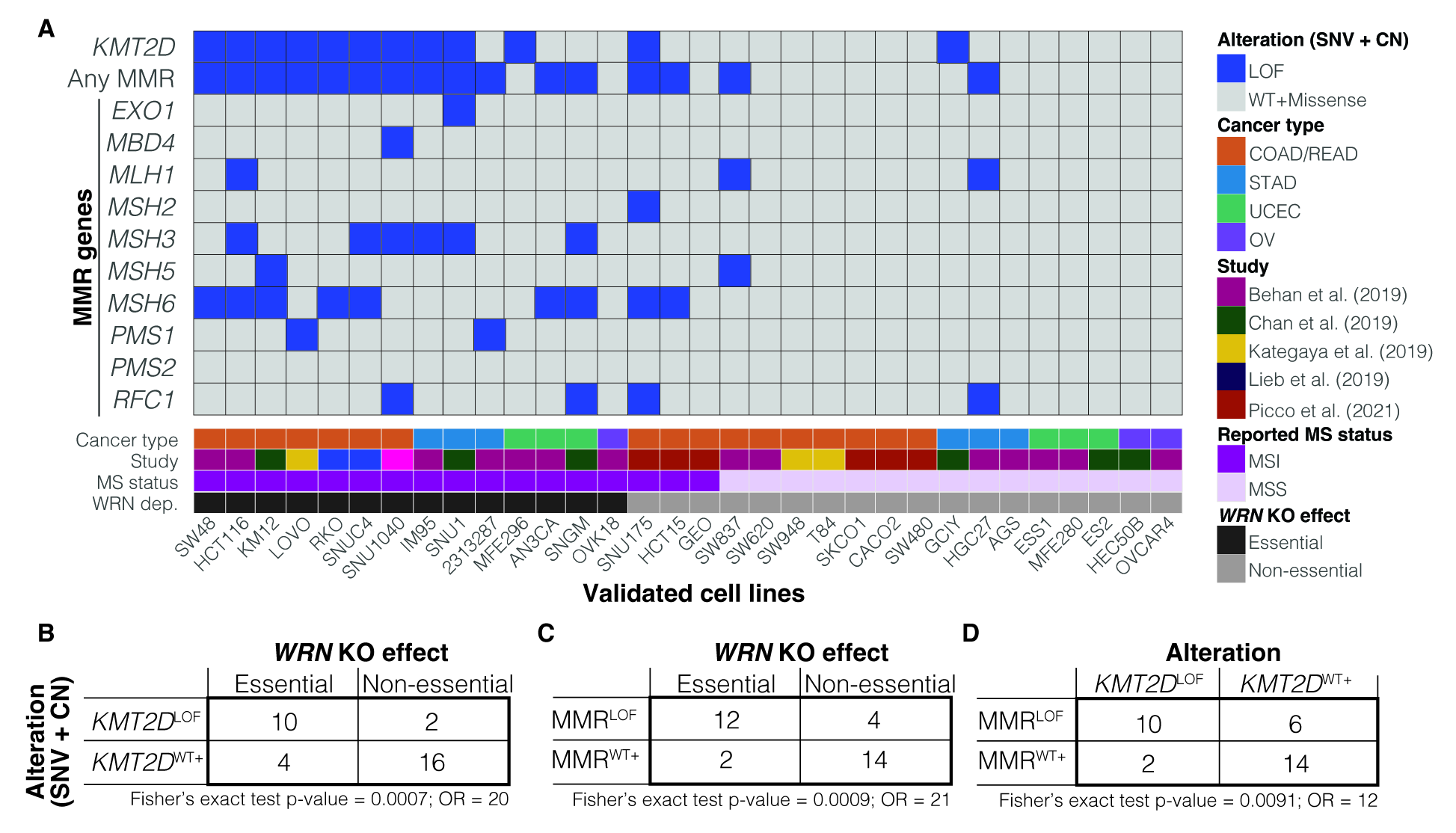

Supplement: Supplementary file 16 — Additional file 16: Fig. S5. MSI cancer cells that require WRN for survival frequently harbour KMT2DLOF mutations. Proportion of advanced and metastatic MSI-IMPACT COAD/READ (left) and all solid cancer (right) cases with MSI and KMT2DLOF alterations. B. Number of cases by cancer type found in the MSK-IMPACT solid cancer cohort (panel A). C. Kaplan–Meier curves (top) and risk table (bottom) comparing overall survival of KMT2DLOF cases to KMT2DWT cases. D. A comparison of TMB between KMT2DWT and KMT2DLOF MSI/MSS cases in MSK-IMPACT COAD/READ and solid cancer cohorts. Welch’s t-test p-value * < 0.05 and *** < 0.001. Groups of less than 3 were not analysed (NA). [file 13073_2024_1401_MOESM16_ESM.tif]
